# Proscan: a structure-based proline design web server

**DOI:** 10.1093/nar/gkae408

**Published:** 2024-05-20

**Authors:** Nathaniel Felbinger, Helder V Ribeiro-Filho, Brian G Pierce

**Affiliations:** University of Maryland Institute for Bioscience and Biotechnology Research, Rockville, MD 20850, USA; Department of Cell Biology and Molecular Genetics, University of Maryland, College Park, MD 20742, USA; University of Maryland Institute for Bioscience and Biotechnology Research, Rockville, MD 20850, USA; Brazilian Biosciences National Laboratory, Brazilian Center for Research in Energy and Materials, Campinas 13083-100, Brazil; University of Maryland Institute for Bioscience and Biotechnology Research, Rockville, MD 20850, USA; Department of Cell Biology and Molecular Genetics, University of Maryland, College Park, MD 20742, USA

## Abstract

The ability to control protein conformations and dynamics through structure-based design has been useful in various scenarios, including engineering of viral antigens for vaccines. One effective design strategy is the substitution of residues to proline amino acids, which due to its unique cyclic side chain can favor and rigidify key backbone conformations. To provide the community with a means to readily identify and explore proline designs for target proteins of interest, we developed the Proscan web server. Proscan provides assessment of backbone angles, energetic and deep learning-based favorability scores, and other parameters for proline substitutions at each position of an input structure, along with interactive visualization of backbone angles and candidate substitution sites on structures. It identifies known favorable proline substitutions for viral antigens, and was benchmarked against datasets of proline substitution stability effects from deep mutational scanning and thermodynamic measurements. This tool can enable researchers to identify and prioritize designs for prospective vaccine antigen targets, or other designs to favor stability of key protein conformations. Proscan is available at: https://proscan.ibbr.umd.edu.

## Graphical Abstract

**Graphical Abstract:**
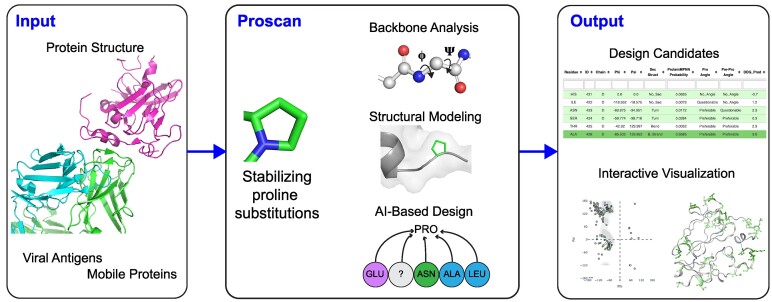

## Introduction

Proline substitutions represent a useful and effective protein design strategy, allowing for favoring and rigidification of key preferred local conformations and tertiary structures due to the proline pyrrolidine ring and consequently constrained main chain dihedral angle. This has been successfully utilized for multiple vaccine antigens to help stabilize prefusion conformations of viral proteins, as discussed in a recent review (1). One notable example is the ‘2P’ coronavirus spike design consisting of two consecutive proline substitutions, which was initially found to stabilize the prefusion state of the MERS-CoV spike glycoprotein (as well as spike glycoproteins from other coronaviruses) in 2017 (2). The 2P design was later utilized to stabilize the SARS-CoV-2 prefusion spike, which enabled determination of the first reported SARS-CoV-2 spike structure (3), and it is used in most approved SARS-CoV-2 vaccines (1). Based on the SARS-CoV-2 spike structure, Hsieh et al. identified a set of four additional proline substitutions to further stabilize the prefusion SARS-CoV-2 spike (4), and this ‘HexaPro’ design (2P plus four more prolines) has been utilized in many subsequent structural and immunogenicity studies (5–8). Examples of proline designs for other viral antigens include the widely used HIV envelope SOSIP design, which contains a key proline residue substitution (I559P)(9), as well as a structure-based proline substitution in the hepatitis C virus (HCV) E2 glycoprotein that led to improved neutralizing antibody binding and immunogenicity (10). Other studies have explored structure-based proline designs to confer improved stability and rigidification in other systems, for instance to stabilize T cell receptor and antibody loop structures to improve antigen binding (11,12).

The success and utility of proline designs in previous studies, in addition to the need for multiple computational tools (10) or large-scale screening strategies required (13), highlights the need for a tool to systematically analyze protein structures to identify candidate proline substitutions. One computational proline design pipeline was reported over 5 years ago (14), but due to its availability as downloadable code, it may not be accessible to researchers with limited computational expertise, while others have proposed a decision tree proline design pipeline (15). Another approach utilized computational mutagenesis in the program Rosetta (16) in conjunction with Ramachandran plot analysis of backbone structure (10), but due to the lack of current availability of the Ramachandran plot web server used in that study (17), and the need to run multiple separate tools in that algorithm, it is not possible to readily utilize it for prospective design studies.

To provide a proline design resource and tool for the community, we have developed the Proscan web server, which includes the Ramachandran plot analysis and Rosetta mutagenesis capabilities previously utilized by our team for antigen design (10), as well as analysis from a recently developed deep learning structure-based design tool, ProteinMPNN (18), and other annotations of interest, including secondary structure and wild-type residue interactions. This easy-to-use server takes protein structures as input, returning results in seconds to minutes (depending on structure size), and its results page includes interactive visualization of results and a formatted table with scores and annotations. Proscan can enable studies to engineer and optimize antigens for current and future vaccine targets, and can be utilized in other design scenarios to stabilize key conformations and modulate protein dynamics.

## Materials and methods

### Web server implementation

#### Interface and visualization

The ProScan web server was developed using the Python3 Flask framework and deployed with Apache. Input Protein Data Bank (PDB)(19) and CIF format files are parsed using a combination of the Biopython PDB package (20) and custom scripts, while PDB file retrieval from PDB code is performed using the Biopython PDB package. Ramachandran plots are rendered using plotly (https://plot.ly), using Ramachandran plot angle distributions from from the MolProbity (21) Top8000 structural database. Protein structures are visualized on the results page with NGL Viewer (22).

#### Structural analysis

Φ and Ψ backbone dihedral angles are calculated for input structures by the Biopython PDB package, and Φ/Ψ angle probability classifications are based on previously established criteria for structural validation (23), with ‘Preferred’, ‘Acceptable’ and ‘Questionable’ referring to within 98%, within 99.5%, and outside of observed proline or pre-proline angle distributions, respectively. Calculations of energetic stability effects for proline substitutions are performed using Rosetta v2.3 (rosettacommons.org) and the ‘interface’ mutagenesis protocol, which structurally models mutant side chains and computes stability changes (ΔΔGs) using an energy-based scoring function (16). Rosetta 2.3 is used in this context due to its speed and its utility in previous design and analysis studies (10,11,24), including strong performance in comparative benchmarking of ΔΔG for alanine point substitutions (25). ProteinMPNN (18) was downloaded from its Github repository (https://github.com/dauparas/ProteinMPNN) in May 2023 and is run on input structures with default parameters and model weights (v_48_020), and positional proline favorability values (0–1, as a proportion to other possible amino acids at each position) are parsed from ProteinMPNN output. Secondary structure information is obtained from the DSSP program (26), hydrogen bonds are determined by the hbplus program (27), and N-glycans are detected by parsing input structures with a script that extracts glycan information from the LINK and struct_connection records of the input PDB/mmCIF file.

### Benchmarking datasets

#### MegaScale dataset

The ΔΔG dataset from the MegaScale deep mutational scanning study (28) was filtered to identify measured values for substitutions to prolines. Sequences of the wild-type proteins were obtained from corresponding PDB structures and clustered with a 30% identity cutoff using MMseqs2 (29), and a PDB was selected from each cluster, excluding NMR structures from selections. This resulted in 16 structures and a total of 652 proline substitutions with measured stability effects.

#### Protherm dataset

An additional dataset of experimentally measured proline mutation ΔΔG values was downloaded from ProthermDB (30). Measured ΔΔG values for proline point substitutions were checked against the corresponding original reference to confirm ΔΔG value and measured polarity. The final curated dataset contains 27 unique proline mutants from nine protein structures.

#### Viral antigens

Viral antigen proline design examples were identified from the literature.

## Results

### Overview of server

**Figure 1.**
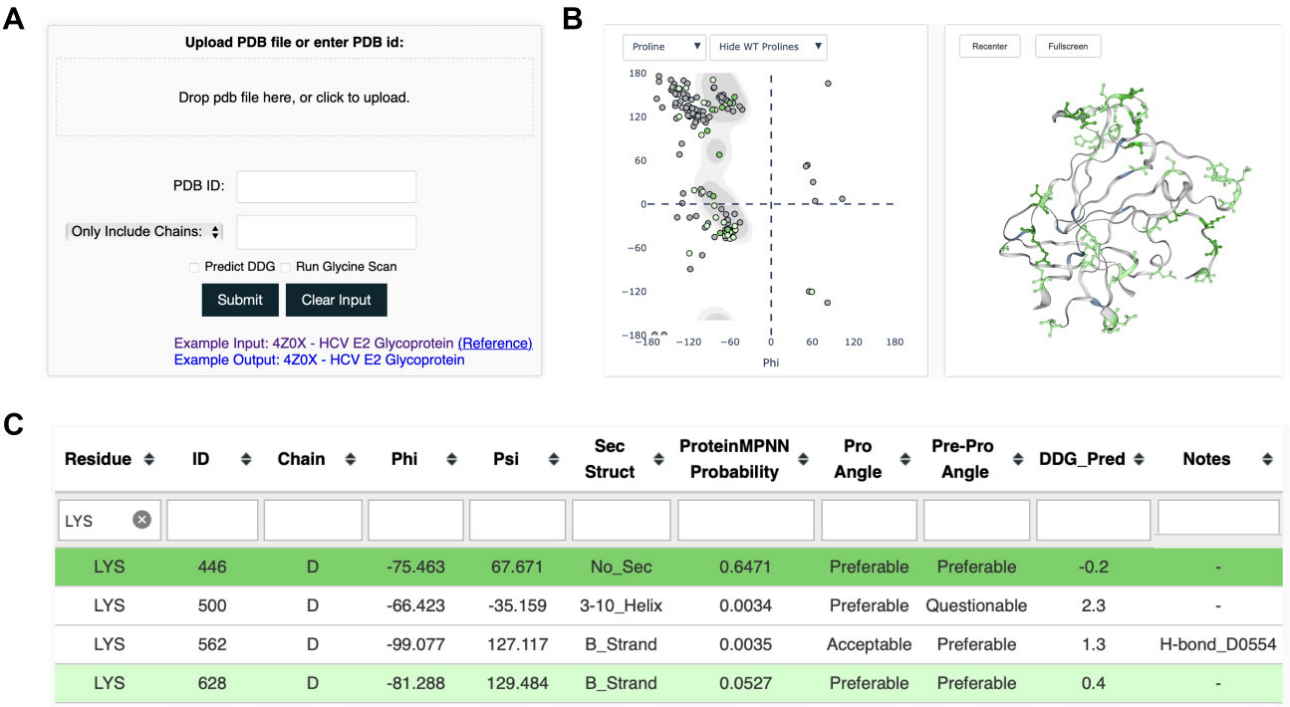
Proscan submission and results pages. (**A**) Proscan submission page, allowing user-input PDB structure or PDB code input, along with user-specified options. (**B**) Results page visualization, with proline Ramachandran plot, showing input structure residue Φ/Ψ backbone angles plotted as points (left), and interactive structure viewer (right). Ramachandran plot points and residues on structure are colored based on predicted proline favorability and score criteria. (**C**) Results page table, showing scores and annotations for each residue and proline substitution favorability. Cells are colored based on score favorability. Example structure shown for (B) and (C) is the HCV E2 glycoprotein core (PDB code 4MWF, chain D) (36). Residues in results table rows and figures are colored by predicted proline favorability (light green = possibly favorable, green = likely favorable).

The Proscan web server takes a protein structure as input, which can be provided through the input page (Figure 1A) as a user-input PDB file, or a PDB code to specify structures. Options to control behavior include the capability to only use certain chains, or exclude certain chains, from analysis, and to skip the Rosetta ΔΔG calculations, which can speed up running time, particularly for larger structures. The Proscan results page (Figure 1B,C) contains a Ramachandran plot showing the distribution of backbone Φ and Ψ angles, a structural viewer with potential proline substitution sites highlighted, and a table with scores and annotations related to proline substitution analysis. The primary scores and data provided by Proscan are Ramachandran plot favorability annotations for proline backbone conformation (current residue) and pre-proline backbone conformation (previous residue), Rosetta stability change for the proline substitution (ΔΔG) in approximate units of kcal/mol, and proline positional probability score from the recently developed deep learning protein design tool ProteinMPNN (18). Rosetta ΔΔG and Ramachandran plot analysis, which primarily represent side chain substitution favorability and backbone compatibility, respectively, have previously been used to select favorable proline designs for viral antigen and other design targets (10–12). The deep learning protein design tool ProteinMPNN uses a graph-based neural network to predict amino acid favorability at each position of a structure, and was shown to be effective at design of structures and protein interfaces (18), as well as protein stability improvement (31). The interactive Proscan output page allows users to download results tables for further analysis, filter the table and viewers based on specific residues, and to filter the table by score criteria and annotations (e.g. secondary structure, hydrogen bond for wild-type residue). For convenience, positions are highlighted based on favorability defined by ProteinMPNN score cutoffs (as noted below), but can be sorted or filtered by users as needed based on additional or other criteria. Proscan running time depends on input protein size, and typically takes seconds to several minutes for most standard sized proteins (<400 residues), while it only takes seconds with Rosetta ΔΔG calculations omitted.

### Benchmarking and score cutoffs

To examine whether Proscan can correctly assess known beneficial proline substitutions, we used Proscan to assess a set of previously published proline substitutions in structures of viral antigen proteins that have high resolution structures of the unmutated proteins available. As shown in Table 1, Proscan favorably assessed the HCV E2, SARS-CoV-2 spike Hexapro, Ebola virus gp, RSV F and Dengue E proline designs based on most metrics. It should be noted that structures containing the designed proline substitutions (except for HCV E2 and Dengue E designs) are within the possible ProteinMPNN training set, however, it is not clear whether the ProteinMPNN model and its output would be biased by those potential instances.

**Table 1.** Proscan assessments of previously described viral antigen proline designs

PDB	Amino acid	Residue number	Chain	Phi	Psi	ProteinMPNN	Ramachandran Proline	Rosetta ΔΔG
*Hepatitis C virus E2 H445P* (10)								
4Z0X	HIS	445	C	−66.4	147.0	0.712	Preferable	−0.6
*SARS-CoV-2 Spike Hexapro* (4)								
6VSB	PHE	817	A	−54.8	−50.7	0.316	Preferable	−0.8
6VSB	ALA	892	A	−63.5	147.8	0.113	Preferable	−1.7
6VSB	ALA	899	A	−58.6	−36.3	0.056	Preferable	−1.2
6VSB	ALA	942	A	−68.3	166.8	0.667	Preferable	−1.3
*RSV F S215P* (37)								
7UJA	SER	215	A	−77.2	−61.34	0.742	Questionable	−0.2
*Ebola gp T577P* (38)								
5JQ3	THR	577	B	−102.3	−7.0	0.015	Preferable	0.6
*Dengue E T280P* (39)								
1TG8	THR	280	A	−59.45	−45.7	0.872	Preferable	−0.9

**Figure 2.**
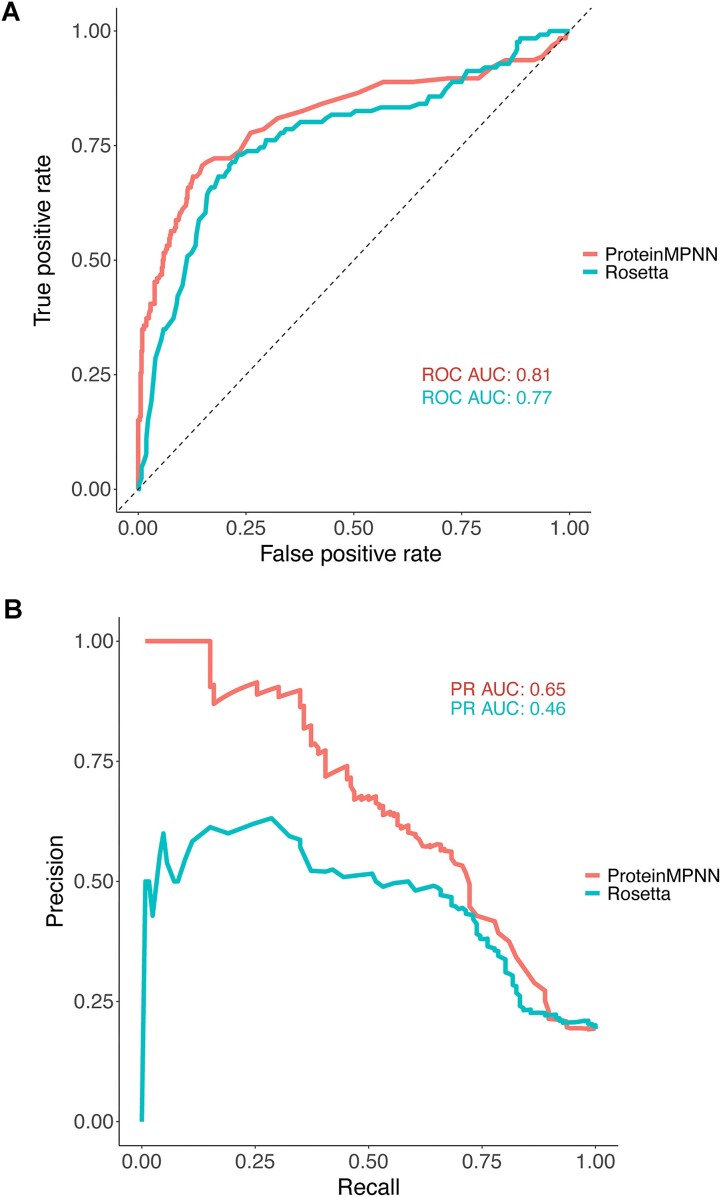
Classification accuracy of ProteinMPNN and Rosetta for proline substitution stability effects from the MegaScale protein stability dataset (28). (**A**) Receiver operating characteristic (ROC) curves for ProteinMPNN and Rosetta scoring of stabilizing versus destabilizing proline substitutions (0.5 kcal/mol ΔΔG cutoff). Numbers of points are 126 (stabilizing) and 526 (destabilizing), and area under curve (AUC) values are shown. (**B**) Precision-recall curves for ProteinMPNN and Rosetta scoring of the same proline substitutions from (A), with AUC values shown.

To more systematically assess the predictive performance of Proscan and its output scores, we used the recently published MegaScale deep mutational scanning dataset (28), from which we obtained a set of over 600 measured proline substitution stability effects (Supplementary Table S1). We examined the performance of scores and criteria to classify substitutions that approximately stabilize or maintain stability (ΔΔG < 0.5) versus those that disrupt stability (ΔΔG > 0.5). Note that the ΔΔG values were negated from original dataset values (28) to align their polarity with most ΔΔG measurements and predictions (positive meaning less favorable), and units are approximately in kcal/mol. As seen in Figure 2A, both Rosetta and ProteinMPNN scores classify stabilizing vs. non-stabilizing substitutions with similar performance based on received operating characteristic (ROC) curve and area under the curve (ROC AUC). Given the somewhat imbalanced dataset (approximately 4:1 destabilizing vs. stabilizing substitutions), we also compared ProteinMPNN and Rosetta with precision-recall curves (Figure 2B), showing that ProteinMPNN is better overall at prediction of proline substitution effects, which is not unexpected given that modeling backbone and other effects from proline substitutions in physics-based modeling programs such as Rosetta can be challenging.

We performed a more detailed analysis of classification accuracies for Ramachandran plot criteria, Rosetta (ΔΔG value cutoff 0.1), and ProteinMPNN (probability score cutoff values 0.2 and 0.01) with the MegaScale dataset (Supplementary Table S2). While backbone angle-based classifications alone (current residue Preferable, or Preferable/Acceptable/No_angle for proline) were effective at identifying some negative (destabilizing) mutations, many false positive (destabilizing) mutations passed the angle filters. Rosetta ΔΔG at the tested cutoff showed some specificity (precision = 0.59) but did not correctly identify the majority of the favorable/neutral substitutions (recall = 0.35). ProteinMPNN performed well in classifying substitutions, with the stricter cutoff (0.2) showing a 1.0 precision value (although detecting only 14% of favorable/neutral substitutions), and the more permissive cutoff (0.01) showing a better balance, with 0.64 precision and 0.56 recall. Combination of the backbone angle-based filters did not markedly improve performance of either Rosetta and ProteinMPNN at the tested score cutoffs. Based on the superior performance of ProteinMPNN in this context, Proscan uses ProteinMPNN score and the two defined score cutoffs (0.2, 0.01) to highlight highly favorable and favorable predicted substitutions, respectively, in the results page table and figures.

To provide additional information regarding the predictive performance of Proscan and its metrics, we identified a set of 27 experimentally measured proline substitution stability changes (ΔΔGs) from the Protherm database (30) (Supplementary Table S3). Computed ProteinMPNN proline probability (log-transformed) and Rosetta ΔΔG values calculated by Proscan with the wild-type structures were found to be highly correlated with the experimentally measured ΔΔGs, with Pearson correlations of 0.69 and 0.66 for ProteinMPNN and Rosetta, respectively (Supplementary Figure S1).

### Example use case

As an example use case for Proscan, we analyzed the antigen chain alone from the HC84.26.5D-E2_434-446_ antibody-antigen complex structure (PDB code 4Z0X) (32), to determine whether the unbound antigen chain without the context of the bound antibody would show favorable scores for the H445P substitution, as we observed for that position in the antibody-antigen complex structure in Table 1. Inputting PDB code 4Z0X, and specifying chain C for ‘Only Include Chains’ to scan the antigen chain alone gives the output results table shown in Supplementary Figure S2. As can be seen, residue 445 is the most favorable position for a proline substitution for the unbound antigen based on ProteinMPNN and Rosetta scores, with ProteinMPNN score of 0.69 (out of 1) and Rosetta ΔΔG of −1.0.

## Discussion

We developed the Proscan server to enable researchers to readily identify and prioritize favorable proline substitutions to stabilize viral antigens and other proteins of interest. Given the range of antigens already successfully engineered with prolines (1), it is quite possible that additional viral and pathogen antigens can be optimized with proline substitutions to favor preferred conformations. Due to current and future emerging pathogens, such design approaches are useful tools in pandemic preparedness (33), as exemplified by the S-2P and Hexapro coronavirus spike designs (4,34), and it is possible that future structure-based antigen designs can be performed from sequence using structural models from accurate deep learning-based modeling methods such as AlphaFold (35).

There are several possible limitations and considerations for Proscan usage. While Proscan is unable to perform multi-state design directly to disfavor certain conformations (e.g. post-fusion antigen conformations) while favoring others (pre-fusion conformation), by downloading Proscan results tables for two or more analyzed structures, the results can be analyzed and compared to effectively address such scenarios. Additionally, while limited structure quality (e.g. resolution > 3.5 Å) may affect Proscan's performance and output utility due to the sensitivity of some of the structure analysis programs, structure refinement can be performed by users to resolve questionable or erroneous geometries prior to Proscan input. While Proscan is expected to prioritize favorable proline substitutions, more detailed simulations of potential dynamic and energetic effects of substitutions can be performed on the top-ranked set of candidates from Proscan for further prioritization.

## Supplementary Material

gkae408_Supplemental_File

## Data Availability

The data underlying this article are available in the article and in its online supplementary material.
