# Supplementary material for: Proscan: a structure-based proline design web server
Source: Nucleic Acids Res. 2024 May 20;52(W1):W280–6. doi: 10.1093/nar/gkae408 (PMC11223860; doi:10.1093/nar/gkae408)
Supplement: gkae408_Supplemental_File [file gkae408_supplemental_file.pdf]

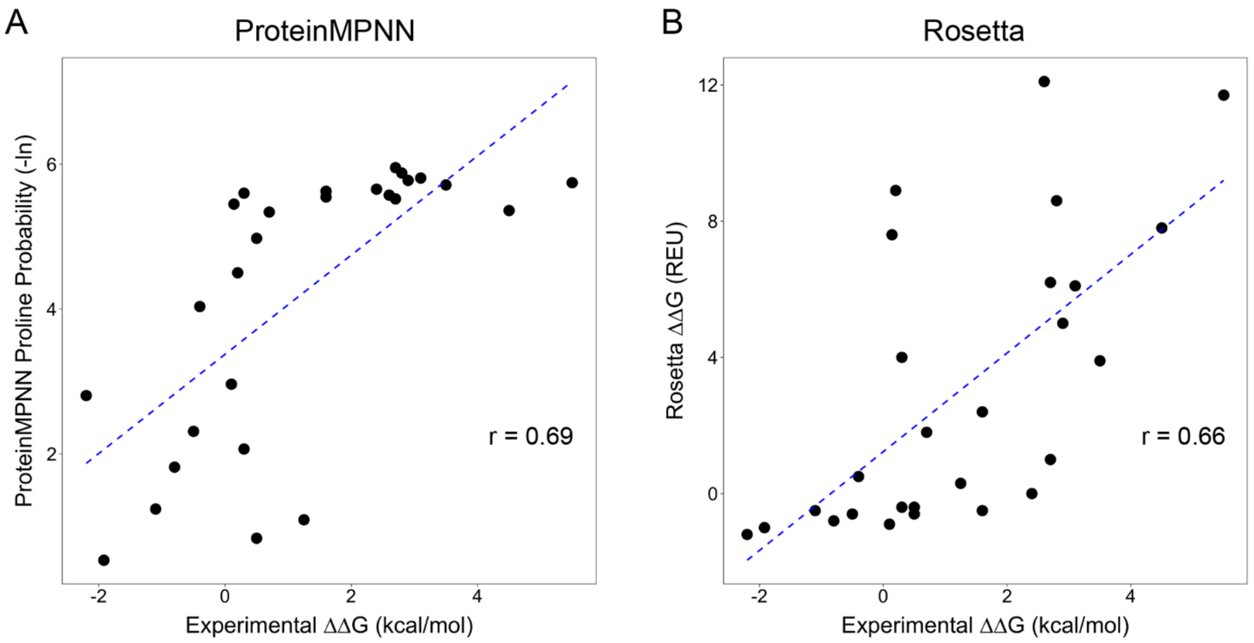

Supplementary Figure 1. Stability effect predictions for a set of ProthermDB (2) proline substitutions. (A) ProteinMPNN probability (negative natural log) and (B) Rosetta ΔΔG values are compared with experimentally measured thermodynamic stability changes (ΔΔG) values for a set of 27 proline substitutions from ProthermDB, with linear fits shown as dotted lines, and Pearson correlations noted. Negative natural log (-ln) of ProteinMPNN probability values is used in this context to convert the probabilities to an approximate scale with Gibbs free energy, while Rosetta ΔΔG values are in Rosetta Energy Units (REU), which are comparable to energy in kcal/mol.

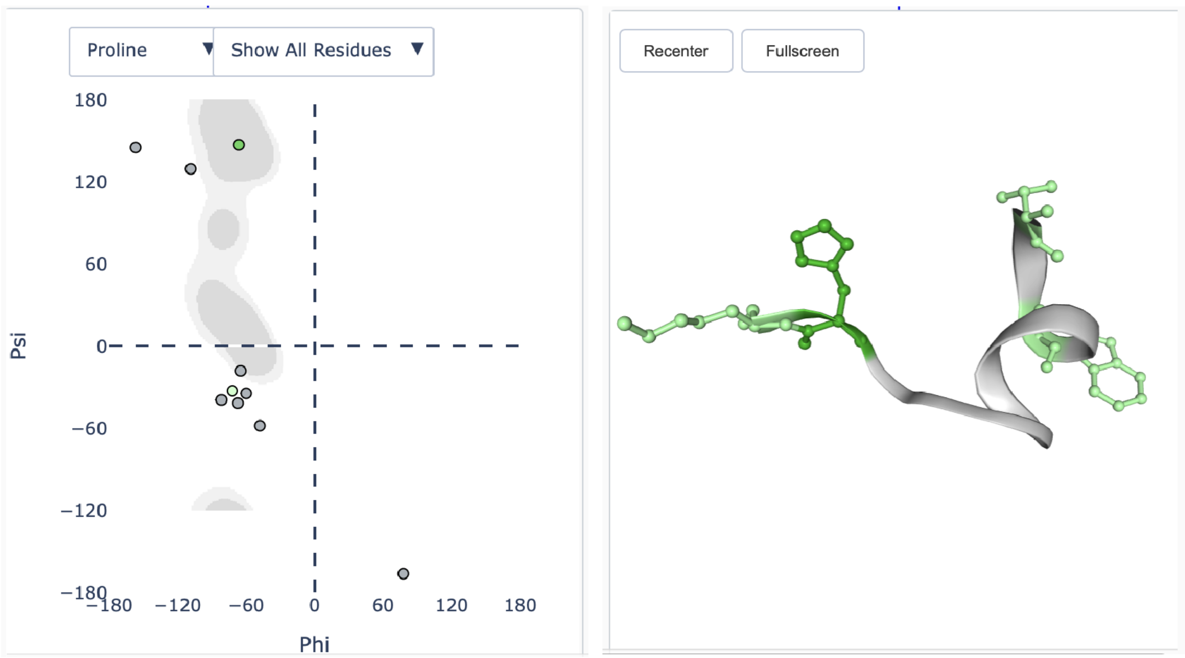

Residue	ID	Chain	Phi	Psi	Sec Struct	ProteinMPNN Probability	Pro Angle	Pre-Pro Angle	DDG_Pred	Notes
THR	435	C	0.0	0.0	No_Sec	0.0188	No_Angle	No_Angle	0.1	-
GLY	436	C	77.615	-166.305	A_Helix	0.0032	Questionable	No_Angle	12.8	-
TRP	437	C	-72.061	-32.674	A_Helix	0.0143	Preferable	Questionable	0.1	-
LEU	438	C	-47.957	-58.172	A_Helix	0.007	Preferable	Preferable	0.0	-
ALA	439	C	-60.082	-34.559	A_Helix	0.0038	Preferable	Preferable	3.2	H-bond_C0435
GLY	440	C	-64.829	-18.146	A_Helix	0.0038	Preferable	Preferable	2.5	H-bond_C0436 H-bond_C0437
LEU	441	C	-67.273	-41.714	A_Helix	0.004	Preferable	Acceptable	8.4	H-bond_C0437 H-bond_C0438
PHE	442	C	-81.694	-39.346	A_Helix	0.0034	Acceptable	Preferable	0.4	H-bond_C0439
TYR	443	C	-156.762	145.016	No_Sec	0.0032	Questionable	Acceptable	3.0	H-bond_C0439
GLN	444	C	-108.53	129.355	No_Sec	0.0036	Questionable	Preferable	1.8	-
HIS	445	C	-66.417	147.011	No_Sec	0.6159	Preferable	Preferable	-1.0	-
LYS	446	C	0.0	0.0	No_Sec	0.1082	No_Angle	No_Angle	0.8	-

Supplementary Figure 2. Proscan results for antigen from HCV antibody-E2 complex (PDB code 4Z0X, chain C). Shown are Ramachandran plot (upper left), antigen structure (upper right) and table of results (bottom), with residues highlighted according to predicted proline substitution favorability (light green = possibly favorable, green = likely favorable).

Supplementary Table 1. Proscan values for MegaScale dataset (1) proline substitutions.

Residue Type	ID	Chain	Phi	Psi	Sec Struct	ProteinMPNN Prob	Pro Angle	Pre-Pro Angle	Rosetta ΔΔG	MegaScale ΔΔG	PDB
SER	23	A	-100.89	139.94	Bend	0.0045	Acceptable	Questionable	1.2	-0.02	1A32
GLU	25	A	-63.1	-44.47	A_Helix	0.0578	Preferable	Preferable	3.7	-0.88	1A32
VAL	26	A	-67.45	-38.53	A_Helix	0.0033	Preferable	Preferable	2.3	-0.8	1A32
GLN	27	A	-66.07	-42.38	A_Helix	0.0029	Preferable	Preferable	7.2	-0.91	1A32
ILE	28	A	-65.25	-38.33	A_Helix	0.0034	Preferable	Preferable	8.9	-1.55	1A32
ALA	29	A	-71.51	-42.95	A_Helix	0.0032	Preferable	Preferable	6.8	-1.03	1A32
ILE	30	A	-61.5	-43.09	A_Helix	0.0036	Preferable	Preferable	7.2	-1.44	1A32
LEU	31	A	-63.55	-46.26	A_Helix	0.0032	Preferable	Preferable	7.6	-1.63	1A32
THR	32	A	-55.73	-46.15	A_Helix	0.0048	Preferable	Preferable	6.6	-1.63	1A32
GLU	33	A	-60.25	-38.25	A_Helix	0.0039	Preferable	Preferable	6.7	-1.43	1A32
GLN	34	A	-75.53	-37.25	A_Helix	0.0033	Preferable	Preferable	5.7	-1.41	1A32
ILE	35	A	-67.41	-36.8	A_Helix	0.0037	Preferable	Preferable	10.2	-1.61	1A32
ASN	36	A	-72.32	-40.24	A_Helix	0.0037	Preferable	Preferable	8.4	-1.39	1A32
ASN	37	A	-66.79	-46.44	A_Helix	0.0038	Preferable	Preferable	7.4	-1.55	1A32
LEU	38	A	-64.02	-33.78	A_Helix	0.0044	Preferable	Preferable	8.6	-1.36	1A32
ASN	39	A	-73.71	-32.55	A_Helix	0.0040	Preferable	Preferable	5.7	-1.39	1A32
GLU	40	A	-74.57	-25.37	A_Helix	0.0041	Preferable	Acceptable	5.3	-1.41	1A32
HIS	41	A	-74.59	-46.39	A_Helix	0.0039	Preferable	Acceptable	9.3	-1.47	1A32
LEU	42	A	-68.2	-20.33	A_Helix	0.0036	Preferable	Preferable	6	-1.4	1A32
ARG	43	A	-62.6	-39.74	A_Helix	0.0033	Preferable	Acceptable	8	-1.16	1A32
VAL	44	A	-102.98	1	A_Helix	0.0040	Preferable	Preferable	0.4	-0.82	1A32
HIS	45	A	-129.02	57.9	No_Sec	0.0023	Questionable	Questionable	7	-1.13	1A32
LYS	46	A	-53.96	-25.26	Turn	0.8093	Preferable	Preferable	-0.4	0.52	1A32
LYS	47	A	-77.14	-13.9	Turn	0.0037	Preferable	Acceptable	1	-0.37	1A32
ASP	48	A	-53.71	152.91	No_Sec	0.0030	Preferable	Questionable	8.8	-0.55	1A32
HIS	49	A	-140.61	-32.54	No_Sec	0.0034	Questionable	Preferable	0.7	-0.18	1A32
HIS	50	A	-70.7	-37.36	A_Helix	0.0040	Preferable	Questionable	1.5	-0.29	1A32
SER	51	A	-76.55	-9.33	A_Helix	0.0056	Preferable	Preferable	6.5	-0.87	1A32
ARG	52	A	-65.14	-48.72	A_Helix	0.0037	Preferable	Questionable	4.6	-0.7	1A32
ARG	53	A	-60.95	-44.92	A_Helix	0.1877	Preferable	Preferable	1.3	-0.9	1A32
GLY	54	A	-59.47	-40.99	A_Helix	0.0121	Preferable	Preferable	-1.3	-0.53	1A32
LEU	55	A	-64.68	-41.17	A_Helix	0.0036	Preferable	Preferable	5.7	-1.63	1A32
LEU	56	A	-63.79	-37.97	A_Helix	0.0037	Preferable	Preferable	3.9	-1.3	1A32
LYS	57	A	-64.93	-42.59	A_Helix	0.0037	Preferable	Preferable	5.2	-1.26	1A32
MET	58	A	-66.33	-39.35	A_Helix	0.0039	Preferable	Preferable	5.4	-1.61	1A32
VAL	59	A	-63.83	-42.1	A_Helix	0.0023	Preferable	Preferable	5.8	-1.77	1A32
GLY	60	A	-67.35	-42.68	A_Helix	0.0034	Preferable	Preferable	4.6	-1.66	1A32
LYS	61	A	-63.44	-30.79	A_Helix	0.0039	Preferable	Preferable	7.2	-1.92	1A32
ARG	62	A	-76.7	-33.08	A_Helix	0.0045	Preferable	Preferable	7.8	-1.78	1A32
ARG	63	A	-64.16	-41.05	A_Helix	0.0039	Preferable	Acceptable	6.3	-2.2	1A32
ARG	64	A	-63.56	-49.73	A_Helix	0.0036	Preferable	Preferable	7	-2.25	1A32
LEU	65	A	-62.36	-41.04	A_Helix	0.0035	Preferable	Preferable	5	-3.2	1A32
LEU	66	A	-64.32	-41.47	A_Helix	0.0038	Preferable	Preferable	9.5	-2.16	1A32
ALA	67	A	-65.36	-34.49	A_Helix	0.0044	Preferable	Preferable	7.9	0.83	1A32
TYR	68	A	-65.11	-51.2	A_Helix	0.0036	Preferable	Preferable	7.9	-2.18	1A32
LEU	69	A	-62.86	-39.26	A_Helix	0.0039	Preferable	Preferable	6.2	-2.54	1A32
ARG	70	A	-57.3	-44.27	A_Helix	0.0034	Preferable	Preferable	7.3	-1.95	1A32
ASN	71	A	-74.85	-37.59	A_Helix	0.0040	Preferable	Preferable	2.9	-1.53	1A32
LYS	72	A	-71.73	-44.27	A_Helix	0.0038	Preferable	Preferable	6.1	-1.35	1A32
ASP	73	A	-158.87	105.75	No_Sec	0.0036	Questionable	Preferable	5.9	-1.68	1A32
VAL	74	A	-62.67	-31.26	A_Helix	0.1819	Preferable	Acceptable	-0.5	0.97	1A32
ALA	75	A	-64.64	-53.99	A_Helix	0.0070	Preferable	Preferable	0.5	-0.38	1A32
ARG	76	A	-63.36	-33.69	A_Helix	0.0034	Preferable	Preferable	8.6	-1.48	1A32
TYR	77	A	-63.51	-50.66	A_Helix	0.0044	Preferable	Preferable	10.8	-1.29	1A32
ARG	78	A	-73.65	-28.65	A_Helix	0.0042	Preferable	Preferable	5.8	-0.99	1A32
GLU	79	A	-65.5	-44.16	A_Helix	0.0034	Preferable	Acceptable	7.6	-1.6	1A32
ILE	80	A	-73.02	-35.83	A_Helix	0.0038	Preferable	Preferable	4.4	-1.69	1A32
VAL	81	A	-66.19	-35.45	A_Helix	0.0039	Preferable	Preferable	4.5	-1.46	1A32
GLU	82	A	-71.35	-42.81	A_Helix	0.0042	Preferable	Preferable	8.8	-1.21	1A32
LYS	83	A	-69.08	-30.69	A_Helix	0.0035	Preferable	Preferable	5.1	-1.23	1A32
LEU	84	A	-91.15	-7.22	A_Helix	0.0043	Preferable	Preferable	5.7	-1.21	1A32
GLY	85	A	69.52	37.15	Turn	0.0039	Questionable	Questionable	2.7	-0.4	1A32
SER	10	A	-83.6	8.82	No_Sec	0.0040	Preferable	Questionable	0.7	0.32	1F0M
PHE	11	A	-123.41	114.83	No_Sec	0.0246	Questionable	Questionable	3.8	-0.59	1F0M
ASN	12	A	-70.09	-14.84	Bend	0.1362	Preferable	Preferable	3.9	-0.43	1F0M
THR	13	A	-136.52	159.8	Bend	0.0041	Questionable	Questionable	7.4	-0.96	1F0M
VAL	14	A	-61.12	-42.88	A_Helix	0.0053	Preferable	Preferable	0.6	-1.84	1F0M
ASP	15	A	-57.13	-39.25	A_Helix	0.0345	Preferable	Preferable	-0.6	-0.23	1F0M
GLU	16	A	-62.79	-51.03	A_Helix	0.0046	Preferable	Preferable	7	-2.44	1F0M
TRP	17	A	-55.37	-48.15	A_Helix	0.0037	Preferable	Preferable	9.6	-3.67	1F0M
LEU	18	A	-59.76	-36.37	A_Helix	0.0043	Preferable	Preferable	8.1	-3.89	1F0M
GLU	19	A	-67.28	-45.55	A_Helix	0.0042	Preferable	Preferable	10	-3.27	1F0M
ALA	20	A	-56.89	-39.71	A_Helix	0.0038	Preferable	Preferable	10.3	-3.88	1F0M
ILE	21	A	-92.01	21.03	Turn	0.0036	Preferable	Preferable	4.7	-3.65	1F0M
LYS	22	A	57.24	24.81	Turn	0.0040	Questionable	Questionable	2.6	-3.01	1F0M
MET	23	A	-108.26	21.16	No_Sec	0.0037	Acceptable	Questionable	12.5	-3.3	1F0M
GLY	24	A	-49.51	-29.99	3-10_Helix	0.0029	Preferable	Questionable	3.9	-0.38	1F0M
GLN	25	A	-54.15	-14.79	3-10_Helix	0.0036	Preferable	Preferable	1.6	-1.31	1F0M
TYR	26	A	-100.66	-9.41	3-10_Helix	0.0031	Preferable	Questionable	8.3	-2.98	1F0M
LYS	27	A	-44.37	-48.44	A_Helix	0.0046	Preferable	Questionable	5.8	-3.26	1F0M
GLU	28	A	-53.24	-47.22	A_Helix	0.1168	Preferable	Preferable	0.3	-0.82	1F0M
SER	29	A	-64.49	-49.41	A_Helix	0.0035	Preferable	Preferable	1.9	-0.49	1F0M

PHE	30	A	-58.4	-51.68	A_Helix	0.0037	Preferable	Preferable	9.2	-3.68	1F0M
ALA	31	A	-54.83	-57.61	A_Helix	0.0040	Preferable	Preferable	6.3	-3.52	1F0M
ASN	32	A	-59.65	-1.13	Turn	0.0043	Preferable	Preferable	4.6	-2.79	1F0M
ALA	33	A	-115.73	4.27	Turn	0.0038	Questionable	Questionable	2.7	-3.21	1F0M
GLY	34	A	87.67	0.83	Turn	0.0037	Questionable	Questionable	7.3	-3.26	1F0M
PHE	35	A	-89.63	74.35	No_Sec	0.0035	Preferable	Questionable	2.9	-3.53	1F0M
THR	36	A	-106.2	4.46	No_Sec	0.0048	Acceptable	Questionable	7.4	-2.81	1F0M
SER	37	A	-141.74	168.76	Bend	0.0040	Questionable	Questionable	1	-3.31	1F0M
PHE	38	A	-78.8	-23.1	A_Helix	0.0038	Preferable	Preferable	2.6	-1.88	1F0M
ASP	39	A	-56.46	-56.42	A_Helix	0.0064	Preferable	Acceptable	2.2	-0.39	1F0M
VAL	40	A	-72.53	-44.69	A_Helix	0.0031	Preferable	Preferable	2.1	-1.85	1F0M
VAL	41	A	-51.41	-44.75	A_Helix	0.0039	Preferable	Preferable	7.1	-3.81	1F0M
SER	42	A	-64.53	-11.7	Turn	0.0037	Preferable	Preferable	7.1	-1.53	1F0M
GLN	43	A	-100.7	-4.23	Turn	0.0033	Preferable	Questionable	4.3	-1.91	1F0M
MET	44	A	-61.9	140.79	No_Sec	0.0036	Preferable	Questionable	12.4	-3.96	1F0M
MET	45	A	-115.84	174.47	No_Sec	0.0041	Questionable	Preferable	5.1	-2.39	1F0M
MET	46	A	-49.19	-46.09	A_Helix	0.0150	Preferable	Preferable	1.5	-0.18	1F0M
GLU	47	A	-48.47	-51.66	A_Helix	0.0106	Preferable	Preferable	0.8	-0.17	1F0M
ASP	48	A	-59.81	-41.53	A_Helix	0.0029	Preferable	Preferable	1.6	-2.79	1F0M
ILE	49	A	-63.63	-39.01	A_Helix	0.0044	Preferable	Preferable	6.6	-3.53	1F0M
LEU	50	A	-67.13	-43.24	A_Helix	0.0040	Preferable	Preferable	6.3	-2.58	1F0M
ARG	51	A	-62.8	-42.94	A_Helix	0.0040	Preferable	Preferable	7.7	-3.12	1F0M
VAL	52	A	-65.2	-7.07	Turn	0.0033	Preferable	Preferable	5.3	-3.84	1F0M
GLY	53	A	94.18	18.49	Turn	0.0033	Questionable	Questionable	8.1	-1.02	1F0M
VAL	54	A	-79.08	110.92	No_Sec	0.0039	Preferable	Questionable	7.8	-3.53	1F0M
THR	55	A	-105.16	-14.74	No_Sec	0.0036	Acceptable	Preferable	2.9	-1.57	1F0M
LEU	56	A	-81.33	128.39	No_Sec	0.0042	Preferable	Questionable	-0.3	-0.73	1F0M
ALA	57	A	-51.87	-48.85	A_Helix	0.1749	Preferable	Preferable	-0.8	0.51	1F0M
GLY	58	A	-61.61	-36.04	A_Helix	0.0066	Preferable	Preferable	-1.3	0.14	1F0M
HIS	59	A	-69.41	-44.4	A_Helix	0.0032	Preferable	Preferable	1.5	-2.13	1F0M
GLN	60	A	-55.11	-46.02	A_Helix	0.0036	Preferable	Preferable	6.8	-2.18	1F0M
LYS	61	A	-64.78	-58.66	A_Helix	0.0042	Preferable	Preferable	5.2	-3.12	1F0M
LYS	62	A	-47.87	-47.57	A_Helix	0.0032	Preferable	Preferable	6.5	-2.28	1F0M
ILE	63	A	-65.75	-51.97	A_Helix	0.0041	Preferable	Preferable	10.1	-4.55	1F0M
LEU	64	A	-56.27	-46.02	A_Helix	0.0037	Preferable	Preferable	8.8	-4.28	1F0M
ASN	65	A	-65.43	-32.36	A_Helix	0.0037	Preferable	Preferable	7.7	-3.55	1F0M
SER	66	A	-69.76	-45.15	A_Helix	0.0032	Preferable	Preferable	7.4	-3.68	1F0M
ILE	67	A	-57.29	-44.43	A_Helix	0.0041	Preferable	Preferable	9.8	-4.06	1F0M
GLN	68	A	-61.48	-35.35	A_Helix	0.0040	Preferable	Preferable	7.4	-2.29	1F0M
VAL	69	A	-70.8	-41.04	A_Helix	0.0036	Preferable	Preferable	4.2	-1.9	1F0M
MET	70	A	-59.25	-54	A_Helix	0.0039	Preferable	Preferable	10.8	-3.04	1F0M
ARG	71	A	-60.5	-44.32	A_Helix	0.0045	Preferable	Preferable	8.7	-2	1F0M
ALA	72	A	-65.69	-35.93	A_Helix	0.0043	Preferable	Preferable	4.8	-1.15	1F0M
GLN	73	A	-69.17	-44.93	A_Helix	0.0042	Preferable	Preferable	9	-0.98	1F0M
MET	74	A	-56.91	-41.94	A_Helix	0.0037	Preferable	Preferable	7.2	-1.07	1F0M
ASN	75	A	-61.7	-25.42	A_Helix	0.0043	Preferable	Preferable	7.5	-0.52	1F0M
LYS	4	A	0	0	No_Sec	0.0727	No_Angle	No_Angle	0.3	0.14	1LP1
PHE	5	A	-58.74	9.45	A_Helix	0.0746	Questionable	No_Angle	5.4	-0.26	1LP1
ASN	6	A	-65.61	-52.47	A_Helix	0.0058	Preferable	Questionable	8.2	0.07	1LP1
LYS	7	A	-68.93	-35.83	A_Helix	0.0078	Preferable	Preferable	1.3	0.1	1LP1
GLU	8	A	-54.81	-53.23	A_Helix	0.0030	Preferable	Preferable	2.7	-0.06	1LP1
LEU	9	A	-63.59	-37.37	A_Helix	0.0042	Preferable	Preferable	7.6	-0.87	1LP1
SER	10	A	-67.02	-49.98	A_Helix	0.0039	Preferable	Preferable	4.6	-0.54	1LP1
VAL	11	A	-71.44	-41.96	A_Helix	0.0039	Preferable	Preferable	7.1	0.03	1LP1
ALA	12	A	-64.1	-46.52	A_Helix	0.0035	Preferable	Preferable	9.8	-1.34	1LP1
GLY	13	A	-55.94	-53.37	A_Helix	0.0039	Preferable	Preferable	11.3	-1.71	1LP1
ARG	14	A	-60.26	-42.26	A_Helix	0.0036	Preferable	Preferable	7.6	-1.42	1LP1
GLU	15	A	-61.2	-44.55	A_Helix	0.0034	Preferable	Preferable	7.2	-1.77	1LP1
ILE	16	A	-62.08	-48.31	A_Helix	0.0024	Preferable	Preferable	8.8	-1.7	1LP1
VAL	17	A	-65.48	-23.72	A_Helix	0.0046	Preferable	Preferable	5.1	-1.47	1LP1
THR	18	A	-89.97	-1.63	Turn	0.0046	Preferable	Acceptable	4.5	-1.91	1LP1
LEU	19	A	-65.27	122.87	No_Sec	0.0044	Preferable	Questionable	6.5	-1.64	1LP1
ASN	21	A	-94.46	-0.7	Turn	0.0041	Preferable	Acceptable	5.2	-2.12	1LP1
LEU	22	A	-89.32	141.13	Bend	0.0040	Preferable	Questionable	1.7	-1.67	1LP1
ASN	23	A	-86.73	168.39	No_Sec	0.0366	Preferable	Preferable	4.3	-1.23	1LP1
ASP	24	A	-53.11	-56.61	A_Helix	0.1040	Preferable	Preferable	0.3	-0.04	1LP1
GLN	26	A	-67.85	-43.51	A_Helix	0.0047	Preferable	Preferable	11	-1.77	1LP1
LYS	27	A	-60.78	-50.2	A_Helix	0.0038	Preferable	Preferable	7	-1.88	1LP1
LYS	28	A	-57.27	-38.99	A_Helix	0.0029	Preferable	Preferable	7.1	-1.22	1LP1
ALA	29	A	-63.17	-49.2	A_Helix	0.0037	Preferable	Preferable	8.2	-1.22	1LP1
PHE	30	A	-59.9	-46.57	A_Helix	0.0036	Preferable	Preferable	8	-2.21	1LP1
ILE	31	A	-59.2	-44.43	A_Helix	0.0037	Preferable	Preferable	9.1	-1.77	1LP1
PHE	32	A	-62.76	-43.76	A_Helix	0.0073	Preferable	Preferable	9.7	-1.7	1LP1
SER	33	A	-63.02	-43.23	A_Helix	0.0036	Preferable	Preferable	6	-1.88	1LP1
LEU	34	A	-53.41	-45.55	A_Helix	0.0040	Preferable	Preferable	11.1	-2.02	1LP1
TRP	35	A	-65.07	-45.69	A_Helix	0.0038	Preferable	Preferable	8.8	-1.48	1LP1
ASP	36	A	-59.3	-30.23	A_Helix	0.0040	Preferable	Preferable	6.4	-2.09	1LP1
ASP	37	A	-145.94	77.21	No_Sec	0.0045	Questionable	Preferable	3.1	-1.45	1LP1
SER	39	A	-54.11	-49.98	3-10_Helix	0.0044	Preferable	Acceptable	4	-0.94	1LP1
GLN	40	A	-98.74	34.06	3-10_Helix	0.0033	Acceptable	Preferable	5.6	-1.31	1LP1
SER	41	A	-55.84	-44.28	A_Helix	0.0035	Preferable	Questionable	2.7	-1.48	1LP1
ALA	42	A	-58.02	-37.74	A_Helix	0.0463	Preferable	Preferable	-0.3	-0.79	1LP1
ASN	43	A	-70.81	-44.36	A_Helix	0.0034	Preferable	Preferable	2.5	-0.92	1LP1
LEU	44	A	-59.59	-47.7	A_Helix	0.0039	Preferable	Preferable	6.3	-1.6	1LP1
LEU	45	A	-57.74	-49.78	A_Helix	0.0036	Preferable	Preferable	6.4	-1.73	1LP1
ALA	46	A	-58.25	-48.4	A_Helix	0.0043	Preferable	Preferable	2.8	-1.9	1LP1
GLU	47	A	-58.81	-41.59	A_Helix	0.0031	Preferable	Preferable	7.7	-2.7	1LP1
ALA	48	A	-65.93	-36.75	A_Helix	0.0036	Preferable	Preferable	8.1	-1.69	1LP1

LYS	49	A	-68.19	-44.48	A_Helix	0.0025	Preferable	Preferable	9.2	-1.8	1LP1
LYS	50	A	-54.62	-47.95	A_Helix	0.0041	Preferable	Preferable	7.3	-1.7	1LP1
LEU	51	A	-65.7	-31.75	A_Helix	0.0035	Preferable	Preferable	4.9	-1.73	1LP1
ASN	52	A	-65.1	-31.66	A_Helix	0.0032	Preferable	Preferable	11.1	-1.91	1LP1
ASP	53	A	-77.43	-40.47	A_Helix	0.0042	Preferable	Preferable	7.5	-1.66	1LP1
ALA	54	A	-62.59	-35.07	A_Helix	0.0045	Preferable	Preferable	4.8	-1.55	1LP1
GLN	55	A	-93.39	10.46	Turn	0.0037	Preferable	Preferable	6.3	-1.5	1LP1
ALA	56	A	-67.77	157.02	Bend	0.0037	Preferable	Questionable	6.7	-0.82	1LP1
LYS	58	A	0	0	No_Sec	0.0416	No_Angle	No_Angle	0	0.09	1LP1
SER	2	A	0	0	No_Sec	0.0256	No_Angle	No_Angle	0.1	0.03	1OPS
GLN	3	A	-86.38	147.12	No_Sec	0.0028	Preferable	No_Angle	1.7	0.04	1OPS
LEU	10	A	-66.15	131.75	No_Sec	0.0311	Preferable	Preferable	-0.2	0.04	1OPS
MET	13	A	-48.98	139.87	Turn	0.0031	Preferable	Preferable	-0.7	-0.37	1OPS
ALA	16	A	-70.04	132.98	B-Bridge	0.2644	Preferable	Preferable	-1.1	-0.12	1OPS
ALA	20	A	-51.59	-21.91	3-10_Helix	0.0056	Preferable	Preferable	-0.5	-0.16	1OPS
GLY	24	A	-96.44	129.81	B_Strand	0.0251	Acceptable	Preferable	-0.2	0.02	1OPS
VAL	26	A	-81.38	118.31	No_Sec	0.0120	Preferable	Preferable	0	-0.04	1OPS
ASN	28	A	-143.81	113.05	Bend	0.0043	Questionable	Preferable	1.7	-4.34	1OPS
ILE	30	A	-82.59	139.98	No_Sec	0.2878	Preferable	Preferable	-0.2	0.04	1OPS
PHE	34	A	-57.02	-24.26	3-10_Helix	0.0037	Preferable	Preferable	10.2	-1.55	1OPS
ALA	35	A	-66.59	-26.2	3-10_Helix	0.0043	Preferable	Acceptable	0.4	-0.29	1OPS
SER	38	A	-60.09	-25.05	3-10_Helix	0.1459	Preferable	Preferable	-1	-0.01	1OPS
GLN	39	A	-81.16	-13.92	3-10_Helix	0.0036	Preferable	Acceptable	1.4	-1.35	1OPS
VAL	41	A	-59.14	125	Turn	0.0044	Preferable	Questionable	1.9	-0.99	1OPS
GLN	44	A	-99.73	153.4	B-Bridge	0.0077	Preferable	Preferable	1	-0.26	1OPS
VAL	45	A	-89.32	140.4	B-Bridge	0.0033	Preferable	Preferable	2.9	-3.52	1OPS
ASN	46	A	-97.72	19.69	Bend	0.0049	Preferable	Preferable	5.8	-4.44	1OPS
ALA	50	A	-78.5	157.25	No_Sec	0.1104	Preferable	Preferable	1.9	-0.23	1OPS
LYS	51	A	-68.21	132.06	Turn	0.0061	Preferable	Preferable	0	0.01	1OPS
THR	54	A	-85.78	128.46	B-Bridge	0.0037	Preferable	Preferable	0.6	-0.61	1OPS
ASN	58	A	-73.12	1.5	3-10_Helix	0.0046	Preferable	Preferable	-0.1	-0.39	1OPS
LYS	61	A	-60.65	120.73	Turn	0.0040	Preferable	Preferable	5.3	-0.78	1OPS
THR	62	A	67.77	19.61	Turn	0.0041	Questionable	Preferable	2.5	-0.07	1OPS
ALA	64	A	-70.77	-14.22	Turn	0.0083	Preferable	Preferable	-1.2	-0.02	1OPS
GLN	3	A	0	0	No_Sec	0.1040	No_Angle	No_Angle	1.2	-0.21	1ORC
ARG	4	A	-73.38	149.65	No_Sec	0.1876	Preferable	No_Angle	0.9	-0.53	1ORC
ILE	5	A	-117.38	138.86	B_Strand	0.0033	Questionable	Preferable	4.4	-2.05	1ORC
THR	6	A	-70.46	159.83	B_Strand	0.0686	Preferable	Preferable	-0.2	0.24	1ORC
LEU	7	A	-59.34	-42.16	A_Helix	0.0048	Preferable	Preferable	4.9	-3.9	1ORC
LYS	8	A	-62.59	-43.88	A_Helix	0.0189	Preferable	Preferable	0.6	-1.06	1ORC
ASP	9	A	-68.46	-34.21	A_Helix	0.0038	Preferable	Preferable	7.3	-4.26	1ORC
TYR	10	A	-63.57	-43.12	A_Helix	0.0040	Preferable	Preferable	8.1	-3.53	1ORC
ALA	11	A	-64.85	-37.08	A_Helix	0.0042	Preferable	Preferable	4	-4.39	1ORC
MET	12	A	-67.83	-31.54	A_Helix	0.0040	Preferable	Preferable	5.7	-3.91	1ORC
ARG	13	A	-82.89	-38.64	A_Helix	0.0044	Acceptable	Preferable	5.8	-4.12	1ORC
PHE	14	A	-113.44	-11.36	A_Helix	0.0037	Questionable	Acceptable	6.6	-4.94	1ORC
GLY	15	A	95.54	154.59	No_Sec	0.0034	Questionable	Questionable	10.1	-3.47	1ORC
GLN	16	A	-68.39	-31.95	A_Helix	0.0175	Preferable	Questionable	1.2	0.03	1ORC
THR	17	A	-66.6	-51.12	A_Helix	0.0282	Preferable	Preferable	0.3	-0.08	1ORC
LYS	18	A	-63.02	-38.35	A_Helix	0.0203	Preferable	Preferable	0.7	-2.59	1ORC
THR	19	A	-57.52	-49.4	A_Helix	0.0066	Preferable	Preferable	5.6	-2.36	1ORC
ALA	20	A	-60.6	-44.46	A_Helix	0.0039	Preferable	Preferable	5.4	-3.48	1ORC
LYS	21	A	-64.24	-44.7	A_Helix	0.0041	Preferable	Preferable	6.5	-3.7	1ORC
ASP	22	A	-62.07	-29.85	A_Helix	0.0047	Preferable	Preferable	6.9	-3.4	1ORC
LEU	23	A	-95.37	-11.36	A_Helix	0.0038	Preferable	Preferable	6.2	-4.11	1ORC
GLY	24	A	67.56	38.87	Turn	0.0044	Questionable	Questionable	2.8	-3.68	1ORC
VAL	25	A	-127.95	170.28	Bend	0.0040	Questionable	Acceptable	3	-2.89	1ORC
TYR	26	A	-94.52	163.43	No_Sec	0.0987	Preferable	Preferable	0.6	0.44	1ORC
GLN	27	A	-56.42	-44.46	A_Helix	0.5145	Preferable	Preferable	1.1	0.28	1ORC
SER	28	A	-53.12	-40.63	A_Helix	0.1505	Preferable	Preferable	-0.3	-0.43	1ORC
ALA	29	A	-66.77	-36.66	A_Helix	0.0042	Preferable	Preferable	2.8	-2.78	1ORC
ILE	30	A	-61.84	-48.58	A_Helix	0.0042	Preferable	Preferable	3.1	-3.89	1ORC
ASN	31	A	-63.86	-36.55	A_Helix	0.0040	Preferable	Preferable	3.1	-0.66	1ORC
LYS	32	A	-70.08	-42.09	A_Helix	0.0032	Preferable	Preferable	5	-1.65	1ORC
ALA	33	A	-56.21	-43.04	A_Helix	0.0025	Preferable	Preferable	5.5	-3.25	1ORC
ILE	34	A	-66.44	-47.73	A_Helix	0.0035	Preferable	Preferable	6.6	-3.63	1ORC
HIS	35	A	-51.61	-46.43	A_Helix	0.0041	Preferable	Preferable	9.4	-2.79	1ORC
ALA	36	A	-72.09	-6.18	Turn	0.0037	Preferable	Preferable	5.8	-2.4	1ORC
GLY	37	A	61.67	39.97	Turn	0.0048	Questionable	Questionable	2.7	-1.72	1ORC
ARG	38	A	-73.42	152.96	No_Sec	0.0038	Preferable	Acceptable	4.8	-3.03	1ORC
LYS	39	A	-95.72	91.3	B_Strand	0.0143	Acceptable	Preferable	0.8	-0.16	1ORC
ILE	40	A	-120.18	130.24	B_Strand	0.0039	Questionable	Acceptable	6.8	-4.78	1ORC
LEU	42	A	-114.81	145.17	B_Strand	0.0042	Questionable	Preferable	6.2	-5.14	1ORC
THR	43	A	-122.24	125.45	B_Strand	0.0045	Questionable	Preferable	3.3	-4.03	1ORC
ILE	44	A	-94.27	129.34	B_Strand	0.0302	Preferable	Preferable	0.8	-1.63	1ORC
ASN	45	A	-93.51	156.22	No_Sec	0.0054	Preferable	Preferable	4.2	-1.55	1ORC
ALA	46	A	-62.69	-19.9	Turn	0.5815	Preferable	Preferable	-0.8	0.18	1ORC
ASP	47	A	-85.91	4.19	Turn	0.0034	Preferable	Acceptable	1	-2.15	1ORC
GLY	48	A	93.25	-14.93	Bend	0.0041	Questionable	Questionable	10.1	-3.94	1ORC
SER	49	A	-78.73	161.83	No_Sec	0.0036	Preferable	Questionable	8.9	-1.83	1ORC
VAL	50	A	-125.63	137.02	B_Strand	0.0034	Questionable	Preferable	2.3	-2.36	1ORC
TYR	51	A	-113.59	129.97	B_Strand	0.0051	Questionable	Preferable	4.2	-4.25	1ORC
ALA	52	A	-121.16	144.41	B_Strand	0.0038	Questionable	Preferable	0.1	-2.58	1ORC
GLU	53	A	-136.64	148.22	B_Strand	0.0043	Questionable	Preferable	2.9	-3.48	1ORC
GLU	54	A	-118.58	142.17	B_Strand	0.0055	Questionable	Preferable	6.1	-3.53	1ORC
VAL	55	A	-104.97	120.42	B_Strand	0.0041	Questionable	Preferable	4.2	-2.51	1ORC

GLU	56	A	-107.73	157.43	B_Strand	0.0053	Acceptable	Questionable	4.6	-2.26	1ORC
ASP	56	A	54.33	53.95	Turn	0.0051	Questionable	Preferable	4.6	-0.97	1ORC
LYS	56	A	-147.92	128.47	B_Strand	0.0050	Questionable	Preferable	4.3	-0.78	1ORC
LYS	56	A	-127.69	161.59	B_Strand	0.0045	Questionable	Preferable	10.5	-2.51	1ORC
VAL	56	A	-109.68	106.51	B_Strand	0.0042	Questionable	Preferable	1.4	-2.33	1ORC
GLY	56	A	71.19	-13.48	Turn	0.0040	Questionable	Preferable	1.9	-1.82	1ORC
PHE	58	A	-129.66	136.51	Bend	0.0040	Questionable	Preferable	3.4	-2.23	1ORC
SER	60	A	-81.64	-51.54	No_Sec	0.1692	Questionable	Preferable	-0.4	-0.12	1ORC
SER	1	A	0	0	No_Sec	0.0252	No_Angle	No_Angle	-0.1	-0.44	1R69
ILE	2	A	-62.07	-34.59	A_Helix	0.0052	Preferable	No_Angle	2.5	-1.9	1R69
SER	3	A	-65.31	-42.47	A_Helix	0.0128	Preferable	Preferable	-0.3	-0.91	1R69
SER	4	A	-73.51	-38.87	A_Helix	0.0047	Preferable	Preferable	0.3	-2.12	1R69
ARG	5	A	-66.85	-33.97	A_Helix	0.0015	Preferable	Preferable	11	-2.6	1R69
VAL	6	A	-72.06	-45.28	A_Helix	0.0033	Preferable	Preferable	9.3	-2.8	1R69
LYS	7	A	-60.63	-51.09	A_Helix	0.0031	Preferable	Preferable	5.9	-3.07	1R69
SER	8	A	-53.16	-46.14	A_Helix	0.0043	Preferable	Preferable	7.1	-2.86	1R69
LYS	9	A	-74.27	-41.55	A_Helix	0.0041	Preferable	Preferable	5.3	-3.1	1R69
ILE	11	A	-64.64	-42.5	A_Helix	0.0041	Preferable	Preferable	5.6	-3.56	1R69
GLN	12	A	-62.61	-28.83	A_Helix	0.0039	Preferable	Preferable	4.4	-3.14	1R69
LEU	13	A	-92.18	-8.39	Turn	0.0038	Preferable	Preferable	4.7	-	1R69
LEU	15	A	-111.26	139.69	No_Sec	0.0042	Questionable	Questionable	5.8	-3.99	1R69
ASN	16	A	-97.44	174.77	No_Sec	0.0073	Preferable	Preferable	6.9	-1.71	1R69
GLN	17	A	-61.68	-36.07	A_Helix	0.0203	Preferable	Preferable	0.8	-0.58	1R69
ALA	18	A	-66.1	-39.69	A_Helix	0.0207	Preferable	Preferable	-0.2	-1.65	1R69
GLU	19	A	-67.93	-39.56	A_Helix	0.0042	Preferable	Preferable	6.5	-3.59	1R69
LEU	20	A	-68.98	-39.79	A_Helix	0.0046	Preferable	Preferable	5.6	-3.5	1R69
ALA	21	A	-59.64	-50.04	A_Helix	0.0035	Preferable	Preferable	6.7	-2.93	1R69
GLN	22	A	-63.8	-34.52	A_Helix	0.0049	Preferable	Preferable	4.1	-3.21	1R69
LYS	23	A	-70.73	-37.42	A_Helix	0.0041	Preferable	Preferable	5.6	-2.67	1R69
VAL	24	A	-80.59	-33.27	A_Helix	0.0037	Preferable	Preferable	7.4	-3.78	1R69
GLY	25	A	88.48	36.64	Turn	0.0037	Questionable	Acceptable	6.5	-0.85	1R69
THR	26	A	-124.2	-173.51	Bend	0.0032	Questionable	Questionable	4.2	-3.34	1R69
THR	27	A	-87.92	157.22	No_Sec	0.0060	Preferable	Acceptable	4.1	-1.4	1R69
GLN	28	A	-62.36	-42.35	A_Helix	0.1531	Preferable	Preferable	0.9	0.01	1R69
GLN	29	A	-58.57	-36.49	A_Helix	0.0905	Preferable	Preferable	1.3	-1.1	1R69
SER	30	A	-65.96	-38.7	A_Helix	0.0057	Preferable	Preferable	5.9	-2.96	1R69
ILE	31	A	-67.27	-41.14	A_Helix	0.0041	Preferable	Preferable	3.3	-2.83	1R69
GLU	32	A	-62.61	-43.96	A_Helix	0.0067	Preferable	Preferable	5.2	-3.29	1R69
GLN	33	A	-66.47	-33.32	A_Helix	0.0026	Preferable	Preferable	6.5	-3.43	1R69
LEU	34	A	-74.62	-38.36	A_Helix	0.0051	Preferable	Preferable	5.9	-3.13	1R69
GLU	35	A	-74.94	-18.04	A_Helix	0.0029	Preferable	Preferable	7.6	-2.86	1R69
ASN	36	A	-102.43	11.27	Turn	0.0043	Preferable	Acceptable	5.8	-3.29	1R69
GLY	37	A	64.17	24.16	Turn	0.0042	Questionable	Questionable	1.9	-2.65	1R69
LYS	38	A	-82.91	-26.33	Bend	0.0046	Preferable	Questionable	4.4	-0.92	1R69
THR	39	A	-132.23	127.64	No_Sec	0.0039	Questionable	Acceptable	1.8	-0.69	1R69
LYS	40	A	-109.57	-20.34	Bend	0.0053	Questionable	Preferable	1.5	0.13	1R69
ARG	41	A	-136.03	67.89	Bend	0.0048	Questionable	Questionable	2.1	-0.78	1R69
ARG	43	A	-72.86	-27.18	Turn	0.1418	Preferable	Preferable	1.1	0.18	1R69
PHE	44	A	-91.29	34.21	Turn	0.0031	Acceptable	Acceptable	3.7	-3.08	1R69
LEU	45	A	-61.47	-44.92	A_Helix	0.0041	Preferable	Questionable	2.2	-3.28	1R69
GLU	47	A	-72.71	-30.82	A_Helix	0.0131	Preferable	Preferable	1	-1.98	1R69
LEU	48	A	-70.73	-42.08	A_Helix	0.0045	Preferable	Acceptable	9.5	-4.07	1R69
ALA	49	A	-62.38	-42.71	A_Helix	0.0034	Preferable	Preferable	4.8	-3.86	1R69
SER	50	A	-66.32	-43.86	A_Helix	0.0037	Preferable	Preferable	3.7	-2.45	1R69
ALA	51	A	-67.58	-27.77	A_Helix	0.0044	Preferable	Preferable	3.8	-	1R69
LEU	52	A	-93.94	-6.56	Turn	0.0044	Preferable	Acceptable	6.5	-3.1	1R69
VAL	54	A	-134.04	172.17	No_Sec	0.0037	Questionable	Acceptable	7	-3.76	1R69
SER	55	A	-88.85	162.46	No_Sec	0.0069	Preferable	Preferable	0	-0.68	1R69
VAL	56	A	-73.69	-36.51	A_Helix	0.0336	Preferable	Preferable	0.9	-0.88	1R69
ASP	57	A	-56.95	-52.57	A_Helix	0.0220	Preferable	Preferable	1.2	-0.91	1R69
TRP	58	A	-54.77	-49.14	A_Helix	0.0033	Preferable	Preferable	12.6	-3.66	1R69
ASN	61	A	-106.44	-29.57	A_Helix	0.0041	Questionable	Preferable	4.6	-0.72	1R69
GLU	1	A	0	0	No_Sec	0.0276	No_Angle	No_Angle	1.5	-0.14	1TG0
VAL	4	A	-60.95	148.87	Bend	0.2089	Preferable	Preferable	-0.4	-0.09	1TG0
LYS	7	A	-87.52	142.99	B_Strand	0.0223	Preferable	Preferable	0.2	-1.96	1TG0
GLN	11	A	-88.98	-22.74	Bend	0.0028	Preferable	Preferable	5.9	-3.07	1TG0
ASP	17	A	-93.31	8.4	Bend	0.0113	Preferable	Preferable	0	0.12	1TG0
TYR	18	A	-102.9	127.28	No_Sec	0.0056	Acceptable	Questionable	1.8	-1.35	1TG0
GLU	19	A	-59.64	-40.91	Turn	0.3904	Preferable	Preferable	0.6	0.92	1TG0
ASP	20	A	-83.41	-2.14	Turn	0.0050	Preferable	Preferable	0	-0.54	1TG0
ASP	21	A	-76	146.45	B-Bridge	0.0040	Preferable	Questionable	8.4	-2.9	1TG0
ASN	23	A	-118.21	156.33	No_Sec	0.0037	Questionable	Preferable	3.3	-0.86	1TG0
GLU	25	A	-92.84	164.47	No_Sec	0.0354	Preferable	Preferable	0.1	-1.15	1TG0
LYS	26	A	-50.06	141.56	Turn	0.0037	Preferable	Preferable	0	-2.14	1TG0
ASP	27	A	83.53	-5.97	Turn	0.0039	Questionable	Preferable	4.9	-2.6	1TG0
GLN	28	A	-64.55	130.34	No_Sec	0.0028	Preferable	Questionable	6.4	-3.35	1TG0
GLU	29	A	-84.72	134.51	B_Strand	0.0089	Preferable	Preferable	1	-1.72	1TG0
ILE	30	A	-132.04	142.42	B_Strand	0.0035	Questionable	Preferable	5.1	-3.24	1TG0
THR	33	A	-112.02	-10.82	B_Strand	0.0038	Questionable	Preferable	2.3	-2.9	1TG0
SER	34	A	-153.13	148.02	B_Strand	0.0032	Questionable	Questionable	1.5	-3.15	1TG0
VAL	35	A	-101.34	111.22	B_Strand	0.0040	Questionable	Preferable	1.4	-2.38	1TG0
GLU	36	A	-79.58	-46.43	No_Sec	0.0035	Acceptable	Preferable	4.3	1	1TG0
ASP	37	A	-165.37	-164.76	Bend	0.0033	Questionable	Preferable	3.8	-1.56	1TG0
ALA	38	A	-66.62	-22.68	Bend	0.0131	Preferable	Questionable	-0.2	0.03	1TG0
GLU	39	A	-102.13	-31.44	Bend	0.0038	Questionable	Acceptable	2.1	-1.44	1TG0
GLY	43	A	-169.57	-166.47	B_Strand	0.0034	Questionable	Preferable	2.9	-3.16	1TG0
GLN	46	A	-96.91	118.91	B_Strand	0.0044	Acceptable	Preferable	1.9	-1.21	1TG0
ASP	47	A	-79.11	-178.14	No_Sec	0.0038	Preferable	Preferable	5	-2.92	1TG0
SER	48	A	-72.17	-20.68	Turn	0.0646	Preferable	Acceptable	-0.1	-0.3	1TG0
ASP	51	A	-92.24	136.89	No_Sec	0.0038	Preferable	Questionable	2.5	-2.91	1TG0

VAL	52	A	-79.38	127.37	B_Strand	0.0070	Preferable	Preferable	0.3	-1.56	1TG0
GLU	54	A	-137.97	159.48	B_Strand	0.0041	Questionable	Preferable	2.1	-2.56	1TG0
GLY	55	A	158.8	-173.08	B_Strand	0.0037	Questionable	Preferable	8.9	-3.17	1TG0
ILE	56	A	-95.59	151.12	B_Strand	0.0035	Preferable	Questionable	3.1	-1.61	1TG0
PHE	57	A	-160.17	158.62	B_Strand	0.0031	Questionable	Preferable	5.4	-1.99	1TG0
LYS	59	A	-54.08	-34.58	3-10_Helix	0.0027	Preferable	Preferable	6	-1.35	1TG0
SER	60	A	-63.42	-18.84	3-10_Helix	0.0047	Preferable	Preferable	-0.2	-2.56	1TG0
ALA	63	A	-124.32	141.83	B_Strand	0.0038	Questionable	Preferable	5.3	-3.21	1TG0
VAL	64	A	-58.92	136.62	B_Strand	0.0423	Preferable	Preferable	-0.3	-0.11	1TG0
GLN	65	A	-76.69	130.2	No_Sec	0.0046	Preferable	Preferable	8.4	0.22	1TG0
GLY	66	A	0	0	No_Sec	0.0063	No_Angle	No_Angle	8.5	0.05	1TG0
ALA	13	A	-49.06	-43.86	A_Helix	0.3280	Preferable	Preferable	-0.3	0.23	2CJJ
LYS	14	A	-78.9	-30.19	A_Helix	0.0056	Preferable	Preferable	0.6	-0.29	2CJJ
ASP	27	A	-71.51	173.8	No_Sec	0.2034	Preferable	Preferable	0.1	0.31	2CJJ
LYS	28	A	-61.48	-19.17	Turn	0.1790	Preferable	Preferable	0	-0.73	2CJJ
ASP	29	A	-84.27	-22.64	Turn	0.0066	Preferable	Acceptable	1.9	-0.77	2CJJ
ALA	35	A	-71.58	-36.58	A_Helix	0.0050	Preferable	Preferable	-0.4	-1.43	2CJJ
GLU	42	A	-55	124.31	Turn	0.5146	Preferable	Preferable	0.1	0.92	2CJJ
GLY	43	A	75.87	43.64	Turn	0.0042	Questionable	Preferable	4.2	-0.75	2CJJ
VAL	57	A	-60.58	-43.28	A_Helix	0.0038	Preferable	Preferable	4.9	-1.16	2CJJ
GLU	58	A	-67.22	-35.43	A_Helix	0.0042	Preferable	Preferable	7.1	-0.41	2CJJ
ASP	59	A	-64.5	-46.28	A_Helix	0.0042	Preferable	Preferable	8.5	-0.4	2CJJ
ILE	60	A	-65.13	-42.81	A_Helix	0.0043	Preferable	Preferable	4.1	0.1	2CJJ
LYS	61	A	-59.34	-46.82	A_Helix	0.0061	Preferable	Preferable	8.2	-0.3	2CJJ
TYR	62	A	-53.37	-52.66	A_Helix	0.0051	Preferable	Preferable	7.6	-0.1	2CJJ
ILE	63	A	-52.97	-43.1	A_Helix	0.0043	Preferable	Preferable	5	-0.1	2CJJ
MET	1	A	0	0	No_Sec	0.0147	No_Angle	No_Angle	6.6	-2.08	2HBB
LYS	2	A	-85.14	130.83	B_Strand	0.0049	Preferable	No_Angle	1.4	-0.88	2HBB
ASP	8	A	-64.34	139.23	No_Sec	0.0089	Preferable	Preferable	0.3	-1.63	2HBB
LYS	10	A	-61.24	127.92	Turn	0.3172	Preferable	Preferable	-0.2	0.68	2HBB
GLY	11	A	92.92	-18.06	Turn	0.0038	Questionable	Preferable	9.4	-2.87	2HBB
LYS	15	A	-46.17	124.44	Turn	0.0033	Preferable	Preferable	5.9	-1.63	2HBB
ASN	20	A	-87.25	140.06	B_Strand	0.0305	Preferable	Preferable	0.3	-0.89	2HBB
ALA	22	A	-59.79	140.55	No_Sec	0.2716	Preferable	Preferable	-1.4	0.52	2HBB
ASP	23	A	-63.36	-41.51	A_Helix	0.0289	Preferable	Preferable	0.7	0.61	2HBB
GLY	24	A	-66.99	-31.47	A_Helix	0.0086	Preferable	Preferable	-0.9	-0.69	2HBB
TYR	25	A	-74.07	-43.6	A_Helix	0.0038	Preferable	Preferable	3.2	-1.35	2HBB
ALA	26	A	-58.11	-52.49	Pi Helix	0.0039	Preferable	Preferable	3.9	-2.19	2HBB
ASN	27	A	-62.42	-46.83	Pi Helix	0.0037	Preferable	Preferable	7.1	-1.67	2HBB
LEU	30	A	-58.47	-53.04	Pi Helix	0.0041	Preferable	Questionable	5.2	-3.12	2HBB
LYS	32	A	-58.47	-38.04	Turn	0.0973	Preferable	Preferable	0.4	0.63	2HBB
LEU	35	A	-91	-22.64	Bend	0.0026	Preferable	Questionable	6.3	-3.01	2HBB
GLU	38	A	-64.11	138.29	B_Strand	0.1984	Preferable	Preferable	-0.3	0.29	2HBB
ALA	39	A	-83.18	49.54	No_Sec	0.0037	Preferable	Preferable	8.1	-2.59	2HBB
THR	40	A	-85.22	155.25	No_Sec	0.0050	Preferable	Questionable	3.5	-2.02	2HBB
ALA	42	A	-59.58	-39.84	A_Helix	0.0132	Preferable	Preferable	-0.7	-1.26	2HBB
ASN	43	A	-73.73	-36.29	A_Helix	0.0028	Preferable	Preferable	7.1	-2.81	2HBB
LEU	44	A	-65.35	-41.71	A_Helix	0.0034	Preferable	Preferable	5.8	-2.71	2HBB
LYS	45	A	-60.51	-51.45	A_Helix	0.0039	Preferable	Preferable	5.2	-1.91	2HBB
ALA	46	A	-60.96	-41.15	A_Helix	0.0037	Preferable	Preferable	4.4	-1.59	2HBB
LEU	47	A	-65.87	-44.81	A_Helix	0.0036	Preferable	Preferable	9.5	-1.74	2HBB
GLU	48	A	-59.78	-39.28	A_Helix	0.0040	Preferable	Preferable	6.8	-0.68	2HBB
ALA	1	A	0	0	No_Sec	0.1076	No_Angle	No_Angle	-0.9	-0.11	2HDZ
GLU	2	A	-56.54	-49.76	A_Helix	0.0060	Preferable	No_Angle	1.4	0.69	2HDZ
GLU	3	A	-57.48	-41.92	A_Helix	0.0485	Preferable	Preferable	0.2	-0.76	2HDZ
ILE	4	A	-64.05	-44.81	A_Helix	0.0066	Preferable	Preferable	0.9	-1.98	2HDZ
GLN	6	A	-58.79	-48.02	A_Helix	0.0029	Preferable	Preferable	9.4	-2.11	2HDZ
GLN	7	A	-59.57	-34.84	A_Helix	0.0045	Preferable	Preferable	6.9	-2.52	2HDZ
SER	8	A	-69.92	-23.37	A_Helix	0.0034	Preferable	Preferable	6.3	-3.7	2HDZ
VAL	9	A	-120.23	-8.3	A_Helix	0.0051	Questionable	Acceptable	4.8	-2.56	2HDZ
ILE	10	A	-57.88	-36	A_Helix	0.0039	Preferable	Questionable	4.2	-3.04	2HDZ
GLY	11	A	-60.5	-50.1	A_Helix	0.0477	Preferable	Preferable	-0.8	-0.9	2HDZ
ASP	12	A	-62.19	-42.53	A_Helix	0.0052	Preferable	Preferable	0.3	-1.42	2HDZ
ALA	15	A	-60.02	-45.96	A_Helix	0.0064	Preferable	Preferable	5.6	-1.1	2HDZ
ARG	16	A	-61.53	-38.25	A_Helix	0.0037	Preferable	Preferable	8	-2.96	2HDZ
LYS	18	A	54.13	44.94	Turn	0.0059	Questionable	Questionable	2.5	-0.71	2HDZ
ASN	19	A	63.85	14.3	Turn	0.0037	Questionable	Acceptable	3.2	-1	2HDZ
ARG	21	A	-61.51	-33.85	A_Helix	0.0052	Preferable	Questionable	1.5	-0.43	2HDZ
VAL	22	A	-68.56	-45.45	A_Helix	0.0168	Preferable	Preferable	0.4	-0.61	2HDZ
LYS	23	A	-65.87	-41.9	A_Helix	0.0037	Preferable	Preferable	11	-1.41	2HDZ
LEU	25	A	-60.93	-45.33	A_Helix	0.0040	Preferable	Preferable	6.9	-3.21	2HDZ
LYS	26	A	-61.16	-43.22	A_Helix	0.0042	Preferable	Preferable	5.3	-2.21	2HDZ
MET	30	A	-64.07	-41.49	A_Helix	0.0050	Preferable	Preferable	5.5	-2.57	2HDZ
THR	31	A	-65.57	-44.74	A_Helix	0.0031	Preferable	Preferable	7.7	-2.46	2HDZ
ASN	33	A	-60.47	-41.83	A_Helix	0.0042	Preferable	Preferable	6.5	-2.05	2HDZ
MET	35	A	-67.68	138.84	Bend	0.0042	Preferable	Questionable	9.4	-3.34	2HDZ
GLU	36	A	-67.3	154.9	No_Sec	0.1405	Preferable	Preferable	-0.1	0.66	2HDZ
LYS	37	A	-54.78	-36.01	A_Helix	0.3375	Preferable	Preferable	0	0.77	2HDZ
LYS	38	A	-65.52	-44.1	A_Helix	0.0084	Preferable	Preferable	0.8	-0.58	2HDZ
GLU	39	A	-66.74	-46.8	A_Helix	0.0059	Preferable	Preferable	1	-2.32	2HDZ
LYS	40	A	-55.65	-41.74	A_Helix	0.0036	Preferable	Preferable	6.8	-3.35	2HDZ
LEU	41	A	-61	-38.58	A_Helix	0.0046	Preferable	Preferable	8.2	-2.32	2HDZ
MET	42	A	-58.89	-44.77	A_Helix	0.0045	Preferable	Preferable	3.5	-0.29	2HDZ
TRP	43	A	-71.76	-30.12	A_Helix	0.0040	Preferable	Preferable	4.2	-1.96	2HDZ
ILE	44	A	-67.57	-44.59	A_Helix	0.0047	Preferable	Acceptable	7.5	-1.92	2HDZ
LYS	45	A	-60.92	-48.34	A_Helix	0.0046	Preferable	Preferable	6.9	-1.23	2HDZ
LYS	46	A	-58.73	-33.95	A_Helix	0.0043	Preferable	Preferable	6	-1.15	2HDZ
ALA	48	A	-94.98	-14.1	A_Helix	0.0042	Preferable	Questionable	5	-1.37	2HDZ

GLU	49	A	-88.66	-12.43	No_Sec	0.0046	Preferable	Questionable	4.3	-0.78	2HDZ
ASP	50	A	0	0	No_Sec	0.0045	No_Angle	No_Angle	0.4	-0.48	2HDZ
ASN	1267	A	-75.04	176.67	No_Sec	0.0094	Preferable	Preferable	0.5	0.09	3DKM
LEU	1268	A	-70.37	-26.39	A_Helix	0.1283	Preferable	Preferable	-0.2	0.56	3DKM
TYR	1269	A	-64.89	-46.08	A_Helix	0.0045	Preferable	Acceptable	2.4	0.27	3DKM
PHE	1270	A	-61.08	-44.92	A_Helix	0.0024	Preferable	Preferable	7.3	0.56	3DKM
GLN	1271	A	-58.77	-45.6	A_Helix	0.0045	Preferable	Preferable	5.8	0.65	3DKM
GLY	1272	A	-51.12	-50.62	A_Helix	0.0034	Preferable	Preferable	7.3	-0.47	3DKM
LEU	1273	A	-59.12	-37.62	A_Helix	0.0030	Preferable	Preferable	5.1	-1.01	3DKM
LYS	1274	A	-78.87	-34.68	A_Helix	0.0027	Preferable	Preferable	7	-0.36	3DKM
TYR	1275	A	-107.06	-20.16	A_Helix	0.0038	Questionable	Acceptable	8.6	-1.18	3DKM
MET	1276	A	-88.9	79.5	No_Sec	0.0025	Preferable	Questionable	8.3	-1.9	3DKM
VAL	1277	A	-133.89	156.14	Bend	0.0035	Questionable	Questionable	7.4	-1.34	3DKM
GLY	1279	A	97.71	-16.4	Turn	0.0037	Questionable	Preferable	11.4	-1.98	3DKM
ALA	1280	A	-63.19	137.02	No_Sec	0.0034	Preferable	Questionable	5.5	-3.34	3DKM
VAL	1282	A	-138.12	158.29	B_Strand	0.0036	Questionable	Preferable	3.2	-2.74	3DKM
THR	1283	A	-123.71	176.57	B_Strand	0.0039	Questionable	Preferable	3	-2.67	3DKM
ARG	1284	A	-60.89	157.53	B_Strand	0.0325	Preferable	Preferable	3	-1.08	3DKM
GLY	1285	A	-135.06	-161.97	No_Sec	0.0035	Questionable	Preferable	23.8	-2.25	3DKM
LEU	1286	A	-55.7	-47.1	Turn	0.3280	Preferable	Questionable	-0.4	0.24	3DKM
ASP	1287	A	-93.45	5.73	Turn	0.0032	Preferable	Preferable	5.4	-1.94	3DKM
TRP	1288	A	-47.07	128.01	No_Sec	0.0030	Preferable	Questionable	12	-2.08	3DKM
LYS	1289	A	-136.31	31.52	No_Sec	0.0026	Questionable	Preferable	3.4	-0.71	3DKM
TRP	1290	A	-112.66	34.52	No_Sec	0.0039	Questionable	Questionable	5.6	-1.14	3DKM
ARG	1291	A	54.02	-135.04	Turn	0.0035	Questionable	Questionable	1.8	-0.56	3DKM
ASP	1292	A	-98.03	31.43	Turn	0.0105	Acceptable	Questionable	2.3	-1.56	3DKM
GLN	1293	A	-68.98	-16.25	Turn	0.0048	Preferable	Questionable	4.7	-1.39	3DKM
ASP	1294	A	-71.87	-9.46	Turn	0.0125	Preferable	Acceptable	4.1	-1.3	3DKM
GLY	1295	A	88.84	-23.76	Bend	0.0032	Questionable	Questionable	7.2	-1.42	3DKM
SER	1296	A	-154.12	157.67	Bend	0.0041	Questionable	Questionable	1.3	-0.54	3DKM
GLN	1298	A	61.22	23.07	Turn	0.0029	Questionable	Preferable	3.2	0.39	3DKM
GLY	1299	A	-61.01	150.67	No_Sec	0.0036	Preferable	Questionable	5.4	-1.53	3DKM
GLU	1300	A	-113.82	162.35	No_Sec	0.0023	Questionable	Preferable	1.9	0.15	3DKM
THR	1302	A	-124.89	135.24	B_Strand	0.0045	Questionable	Preferable	3.8	-3.06	3DKM
GLU	1306	A	-101.59	163.51	No_Sec	0.0067	Preferable	Questionable	1.1	0.01	3DKM
LEU	1307	A	-59.15	141.73	No_Sec	0.0067	Preferable	Preferable	0.9	-0.74	3DKM
HIS	1308	A	-140.49	114.15	B-Bridge	0.0032	Questionable	Preferable	3.8	-2.36	3DKM
ASN	1309	A	52.02	32.05	Turn	0.0036	Questionable	Preferable	3	-2.36	3DKM
ASP	1313	A	-88.88	139.13	B_Strand	0.0221	Preferable	Preferable	0.8	0.06	3DKM
ALA	1318	A	-69.28	-15.9	Turn	0.0044	Preferable	Preferable	-1.2	-3.14	3DKM
GLY	1320	A	-79.42	148.67	No_Sec	0.0050	Preferable	Questionable	-2	-1.08	3DKM
SER	1321	A	-118.02	132.53	B_Strand	0.0036	Questionable	Preferable	1.3	-2.34	3DKM
ASN	1322	A	-159.14	-173.92	B_Strand	0.0032	Questionable	Preferable	2.8	-2.8	3DKM
SER	1323	A	-123.82	145.23	B_Strand	0.0029	Questionable	Acceptable	4.5	-1.47	3DKM
ARG	1325	A	-80.76	131.47	B_Strand	0.0301	Preferable	Preferable	11.5	-3.24	3DKM
ALA	1328	A	-57.51	141.34	3-10_Helix	0.0033	Preferable	Questionable	4.5	-2.86	3DKM
LYS	1331	A	-119.26	150.84	No_Sec	0.0018	Questionable	Questionable	8.1	-3.4	3DKM
ASP	1333	A	-91.23	-9.52	No_Sec	0.0033	Preferable	Preferable	11.3	-3.06	3DKM
LEU	1334	A	-123.35	161.43	B_Strand	0.0038	Questionable	Questionable	3.7	-3.16	3DKM
LEU	1336	A	-61.35	141.63	B_Strand	0.0534	Preferable	Preferable	1.2	-0.57	3DKM
ALA	1337	A	-71.07	157.03	Poly_Proline	0.0038	Preferable	Preferable	6.4	-1.6	3DKM
ASP	1227	A	-91.34	27.32	No_Sec	0.0321	Acceptable	No_Angle	1.6	-0.19	3L1X
ALA	1228	A	-61.43	134.74	No_Sec	0.0040	Preferable	Questionable	2.9	0.34	3L1X
ASP	1230	A	-51.84	-39.17	3-10_Helix	0.1221	Preferable	Preferable	-0.1	0.86	3L1X
GLU	1231	A	-61.36	-18.04	3-10_Helix	0.0045	Preferable	Preferable	0.7	-0.85	3L1X
ARG	1233	A	-99.88	147.49	B-Bridge	0.0038	Acceptable	Questionable	7.4	-3.01	3L1X
ASP	1234	A	-69.56	131.13	No_Sec	0.0028	Preferable	Preferable	10.2	-3.39	3L1X
ASP	1238	A	50.81	22.57	No_Sec	0.0046	Questionable	Questionable	2.2	-2.73	3L1X
LEU	1240	A	-63.64	127.63	B-Bridge	0.0072	Preferable	Preferable	1.2	-1.92	3L1X
THR	1242	A	-113.77	-12.55	Bend	0.0043	Questionable	Preferable	2	-2.33	3L1X
THR	1251	A	-69.72	134.42	No_Sec	0.0047	Preferable	Questionable	4.5	-1.5	3L1X
SER	1256	A	-55.82	-47.97	A_Helix	0.0160	Preferable	Preferable	-0.3	-0.33	3L1X
THR	1267	A	-141.99	160.92	B-Bridge	0.0045	Questionable	Questionable	9.3	-2.9	3L1X
ASP	1268	A	-71.39	125.35	No_Sec	0.0066	Preferable	Preferable	7.4	-3.4	3L1X
ARG	1272	A	75.18	1.11	No_Sec	0.0050	Questionable	Questionable	3.7	-1.85	3L1X
GLN	1273	A	-65.99	155.22	No_Sec	0.0051	Preferable	Questionable	8.2	-3.06	3L1X
THR	1274	A	-66.76	141.3	B-Bridge	0.4049	Preferable	Preferable	-0.6	0.94	3L1X
GLU	1277	A	-50.17	-46.03	3-10_Helix	0.3615	Preferable	Preferable	1.2	0.05	3L1X
SER	1278	A	-54.94	-21.47	3-10_Helix	0.0186	Preferable	Preferable	-0.4	-1.17	3L1X
VAL	1283	A	-113	80.58	No_Sec	0.0055	Questionable	Preferable	4.9	-3.34	3L1X
GLU	1285	A	-59.3	-46.17	A_Helix	0.0305	Preferable	Preferable	1.1	-1.9	3L1X
GLN	1291	A	-59.16	-41.11	A_Helix	0.0040	Preferable	Preferable	6.9	-2.28	3L1X
MET	1294	A	-74.44	-48.75	A_Helix	0.0035	Acceptable	Preferable	8.3	-2.61	3L1X
ARG	1295	A	-60.97	-25.72	A_Helix	0.0042	Preferable	Preferable	7	-1.68	3L1X
GLY	1	A	-80.95	1.57	Turn	0.0054	Preferable	Questionable	0	-0.58	3V1A
SER	2	A	-65.84	136.53	Bend	0.0059	Preferable	Questionable	0.1	-0.71	3V1A
LEU	4	A	-58.07	-41.6	A_Helix	0.0040	Preferable	Preferable	3.6	-1.9	3V1A
ALA	5	A	-58.27	-41.64	A_Helix	0.0033	Preferable	Preferable	3.9	-1.19	3V1A
GLN	6	A	-69.16	-35.31	A_Helix	0.0043	Preferable	Preferable	3	-1.87	3V1A
GLN	7	A	-58.67	-41.65	A_Helix	0.0031	Preferable	Preferable	6.6	-2.3	3V1A
ILE	8	A	-60.49	-47.42	A_Helix	0.0048	Preferable	Preferable	6.5	-3.33	3V1A
LYS	9	A	-56.37	-39.44	A_Helix	0.0031	Preferable	Preferable	3.3	-2.64	3V1A
ASN	10	A	-64.18	-41.43	A_Helix	0.0038	Preferable	Preferable	7.7	-2.54	3V1A
ILE	11	A	-61.58	-43.53	A_Helix	0.0035	Preferable	Preferable	6.4	-3.28	3V1A
HIS	12	A	-54.6	-44.39	A_Helix	0.0037	Preferable	Preferable	4.7	-2.85	3V1A
SER	13	A	-61.18	-44.78	A_Helix	0.0039	Preferable	Preferable	5.2	-2.37	3V1A

PHE	14	A	-62.22	-36.34	A_Helix	0.0036	Preferable	Preferable	4.5	-3.73	3V1A
HIS	16	A	-59.35	-46.76	A_Helix	0.0040	Preferable	Preferable	4.8	-2.74	3V1A
GLN	17	A	-62.88	-36.59	A_Helix	0.0040	Preferable	Preferable	6.6	-2.8	3V1A
ALA	18	A	-70.07	-40.7	A_Helix	0.0040	Preferable	Preferable	6	-3.09	3V1A
LYS	19	A	-59.08	-38.89	A_Helix	0.0038	Preferable	Preferable	7.7	-2.7	3V1A
ALA	20	A	-61.92	-20.03	Turn	0.0036	Preferable	Preferable	5.3	-2.56	3V1A
ALA	21	A	-107.05	7.09	Turn	0.0044	Acceptable	Acceptable	0	-2.6	3V1A
GLY	22	A	73.69	19.87	Turn	0.0040	Questionable	Questionable	3.9	-2.64	3V1A
ARG	23	A	-83.22	70.93	No_Sec	0.0046	Preferable	Questionable	5.2	-2.61	3V1A
MET	24	A	-69.31	-37.96	A_Helix	0.0062	Preferable	Questionable	4.3	-1.31	3V1A
ASP	25	A	-57.9	-47.07	A_Helix	0.0109	Preferable	Preferable	0.3	-1.88	3V1A
GLU	26	A	-67.68	-39.94	A_Helix	0.0040	Preferable	Preferable	0.6	-2.32	3V1A
VAL	27	A	-56.21	-47.51	A_Helix	0.0043	Preferable	Preferable	7.4	-2.67	3V1A
ARG	28	A	-62.94	-39.58	A_Helix	0.0038	Preferable	Preferable	6.5	-1.93	3V1A
THR	29	A	-65.19	-47.15	A_Helix	0.0036	Preferable	Preferable	3.2	-2.02	3V1A
LEU	30	A	-62.86	-32.66	A_Helix	0.0053	Preferable	Preferable	7.3	-3.07	3V1A
GLN	31	A	-59.32	-43.45	A_Helix	0.0036	Preferable	Preferable	8.8	-2.6	3V1A
GLU	32	A	-60.22	-45.52	A_Helix	0.0040	Preferable	Preferable	3.1	-2.47	3V1A
ASN	33	A	-61.88	-43.6	A_Helix	0.0036	Preferable	Preferable	4	-2.3	3V1A
LEU	34	A	-52.87	-52.97	A_Helix	0.0049	Preferable	Preferable	11.7	-3.93	3V1A
HIS	35	A	-64.94	-35.84	A_Helix	0.0040	Preferable	Preferable	5.9	-2.96	3V1A
GLN	36	A	-64.04	-43.88	A_Helix	0.0040	Preferable	Preferable	7.6	-2.3	3V1A
LEU	37	A	-68.61	-37.11	A_Helix	0.0039	Preferable	Preferable	5.2	-3.89	3V1A
MET	38	A	-59.4	-43.31	A_Helix	0.0028	Preferable	Preferable	8.1	-3.54	3V1A
HIS	39	A	-68.49	-37.12	A_Helix	0.0040	Preferable	Preferable	5.8	-2.5	3V1A
GLU	40	A	-58.85	-48.44	A_Helix	0.0033	Preferable	Preferable	5.5	-1.95	3V1A
TYR	41	A	-58.6	-47.28	A_Helix	0.0036	Preferable	Preferable	9.4	-2.2	3V1A
PHE	42	A	-82.6	-12.01	A_Helix	0.0042	Preferable	Preferable	5.5	-1.95	3V1A
GLN	43	A	-90.99	-42.71	A_Helix	0.0031	Questionable	Questionable	7.9	-1.21	3V1A
GLN	44	A	-82.36	127.02	Bend	0.0029	Preferable	Acceptable	7.2	-1.13	3V1A
PHE	876	A	-82.88	-21.66	A_Helix	0.0144	Preferable	Preferable	4.4	-0.32	5JRT
SER	877	A	-64.56	-37.26	A_Helix	0.0079	Preferable	Acceptable	-0.1	-0.56	5JRT
ILE	878	A	-66.27	-39.53	A_Helix	0.0042	Preferable	Preferable	3.9	-2.15	5JRT
THR	879	A	-57.33	-45.86	A_Helix	0.0037	Preferable	Preferable	7.8	-0.82	5JRT
GLN	880	A	-62.64	-45.13	A_Helix	0.0044	Preferable	Preferable	7.2	-1.66	5JRT
PHE	881	A	-60.95	-45.96	A_Helix	0.0040	Preferable	Preferable	7.3	-3.52	5JRT
VAL	882	A	-71.39	-39.37	A_Helix	0.0042	Preferable	Preferable	5.8	-3.31	5JRT
ARG	883	A	-60.53	-40.1	A_Helix	0.0040	Preferable	Preferable	3	-3.01	5JRT
ASN	884	A	-62.46	-30.43	A_Helix	0.0037	Preferable	Preferable	2.7	-1.98	5JRT
LEU	885	A	-82.94	-4.28	Turn	0.0038	Preferable	Preferable	6.3	-3.18	5JRT
GLY	886	A	68.73	28.71	Turn	0.0040	Questionable	Questionable	-0.1	-2.42	5JRT
LEU	887	A	-112.47	28	No_Sec	0.0039	Questionable	Questionable	0.3	-2.54	5JRT
GLU	888	A	-57.01	-24.07	3-10_Helix	0.0050	Preferable	Questionable	7	-0.46	5JRT
HIS	889	A	-63.15	-20.39	3-10_Helix	0.0050	Preferable	Acceptable	9.6	-0.8	5JRT
LEU	890	A	-86.18	-6.9	3-10_Helix	0.0026	Preferable	Acceptable	0.4	-3.61	5JRT
MET	891	A	-58.82	-29.11	A_Helix	0.0042	Preferable	Questionable	6.5	-2.57	5JRT
ASP	892	A	-60.29	-37.23	A_Helix	0.7525	Preferable	Preferable	4.9	0.42	5JRT
ILE	893	A	-71.72	-51.33	A_Helix	0.0041	Acceptable	Preferable	8.1	-2.56	5JRT
PHE	894	A	-57.85	-39.64	A_Helix	0.0029	Preferable	Preferable	6.3	-2.71	5JRT
GLU	895	A	-67.39	-48.14	A_Helix	0.0040	Preferable	Preferable	6.8	-2	5JRT
ARG	896	A	-61.5	-34.29	A_Helix	0.0033	Preferable	Preferable	2.9	-1.67	5JRT
GLU	897	A	-89.01	1.18	Turn	0.0036	Preferable	Preferable	7.2	-1.81	5JRT
GLN	898	A	56.24	44.35	Turn	0.0037	Questionable	Questionable	0.3	-1.43	5JRT
ILE	899	A	-88.17	121.81	No_Sec	0.0040	Preferable	Acceptable	9.1	-1.93	5JRT
THR	900	A	-97.6	167.84	No_Sec	0.0042	Preferable	Preferable	0.5	-1.41	5JRT
LEU	901	A	-61.23	-40.44	A_Helix	0.0042	Preferable	Preferable	5.4	-1.71	5JRT
ARG	902	A	-59.65	-37.76	A_Helix	0.0072	Preferable	Preferable	7.3	-0.27	5JRT
VAL	903	A	-72.01	-46.02	A_Helix	0.0049	Preferable	Preferable	5	-2.41	5JRT
LEU	904	A	-53.89	-38.61	A_Helix	0.0043	Preferable	Preferable	0.9	-3.1	5JRT
VAL	905	A	-55.51	-31.11	Turn	0.0049	Preferable	Preferable	7.2	-1.43	5JRT
GLU	906	A	-93.19	2.23	Turn	0.0038	Preferable	Preferable	1.7	-0.54	5JRT
MET	907	A	-93	128.13	No_Sec	0.0032	Preferable	Questionable	3	-2.61	5JRT
GLY	908	A	-106.44	-167.18	No_Sec	0.0032	Questionable	Preferable	0.6	-2.43	5JRT
HIS	909	A	-54.74	-43.58	A_Helix	0.0346	Preferable	Questionable	1.8	-1.83	5JRT
LYS	910	A	-57.07	-51.98	A_Helix	0.0203	Preferable	Preferable	8.7	-0.48	5JRT
GLU	911	A	-66.76	-41.64	A_Helix	0.0083	Preferable	Preferable	7.9	-2.07	5JRT
LEU	912	A	-69.74	-37.79	A_Helix	0.0043	Preferable	Preferable	6.9	-2.76	5JRT
LYS	913	A	-64.63	-36.62	A_Helix	0.0044	Preferable	Preferable	2.6	-2.42	5JRT
GLU	914	A	-57.18	-39.65	A_Helix	0.0037	Preferable	Preferable	9.1	-2.67	5JRT
ILE	915	A	-88.13	2.89	Turn	0.0028	Preferable	Preferable	4.7	-3.07	5JRT
GLY	916	A	97.1	19.51	Turn	0.0035	Questionable	Questionable	2.1	-2.68	5JRT
ILE	917	A	-88.56	86.95	No_Sec	0.0036	Acceptable	Questionable	-0.8	-2.08	5JRT
ASN	918	A	-74.09	-28.89	No_Sec	0.0037	Preferable	Acceptable	0.4	-0.72	5JRT
ALA	919	A	-87.52	124.8	No_Sec	0.0040	Preferable	Acceptable	-1.2	-0.75	5JRT
TYR	920	A	-53.43	-43.39	A_Helix	0.0779	Preferable	Preferable	2.3	-0.45	5JRT
GLY	921	A	-61.42	-35.27	A_Helix	0.0052	Preferable	Preferable	9.4	-0.18	5JRT
HIS	922	A	-70.35	-40.99	A_Helix	0.0039	Preferable	Preferable	7.7	-0.53	5JRT
ARG	923	A	-65.52	-42.26	A_Helix	0.0053	Preferable	Preferable	7.7	-2.22	5JRT
GLU	924	A	-61.87	-39.31	A_Helix	0.0042	Preferable	Preferable	7.8	-2.48	5JRT
LYS	925	A	-62.84	-40	A_Helix	0.0028	Preferable	Preferable	8.1	-2.52	5JRT
LEU	926	A	-69.88	-49.45	A_Helix	0.0038	Preferable	Preferable	8.2	-2.96	5JRT
ILE	927	A	-61.35	-44.46	A_Helix	0.0038	Preferable	Preferable	11.6	-2.44	5JRT
LYS	928	A	-71.55	-30.4	A_Helix	0.0039	Preferable	Preferable	7.1	-2.45	5JRT
GLY	929	A	-63.77	-39.25	A_Helix	0.0038	Preferable	Acceptable	5.2	-2.72	5JRT
VAL	930	A	-68.98	-46.02	A_Helix	0.0050	Preferable	Preferable	7.4	-2.81	5JRT
GLU	931	A	-55.94	-43.98	A_Helix	0.0037	Preferable	Preferable	7.9	-1.83	5JRT
ARG	932	A	-72.18	-35.18	A_Helix	0.0041	Preferable	Preferable	7.1	-1.81	5JRT
LEU	933	A	-62.28	-43.2	A_Helix	0.0036	Preferable	Preferable	7.8	-2.43	5JRT

ILE	934	A	-68.6	-50.74	A_Helix	0.0040	Preferable	Preferable	9.1	-0.96	5JRT
GLY	8	A	-62.87	-42.97	A_Helix	0.0714	Preferable	Preferable	10.1	0.9	5Z2S
ARG	22	A	-75.16	-19.83	Bend	0.5362	Preferable	Preferable	-1.1	0.58	5Z2S
GLY	25	A	-75.75	177.79	No_Sec	0.0051	Preferable	Preferable	-0.3	-0.7	5Z2S
ILE	26	A	-62.02	-38.06	A_Helix	0.0532	Preferable	Preferable	-0.4	0.25	5Z2S
ALA	27	A	-64.12	-42.47	A_Helix	0.0243	Preferable	Preferable	10.4	-1.41	5Z2S
GLU	40	A	-56.13	-40.06	A_Helix	0.0171	Preferable	Preferable	3.4	-0.45	5Z2S
SER	41	A	-62.04	-32.74	A_Helix	0.0126	Preferable	Preferable	6.5	-0.35	5Z2S
ARG	339	A	-52.24	-35.51	Turn	0.1257	Preferable	Preferable	0.8	0.1	7JJK
MET	341	A	-65.16	-38.75	A_Helix	0.0035	Preferable	Preferable	8.3	-0.01	7JJK
ASN	342	A	-68.74	-37.75	A_Helix	0.0137	Preferable	Preferable	0.3	-1.36	7JJK
ALA	343	A	-66.59	-44.22	A_Helix	0.0043	Preferable	Preferable	0	-0.92	7JJK
MET	345	A	-66.1	-30.84	A_Helix	0.0034	Preferable	Preferable	7.4	-2.09	7JJK
ARG	349	A	-59.18	-36.06	A_Helix	0.0038	Preferable	Preferable	7.2	-2.07	7JJK
ALA	354	A	-71.69	-35.02	A_Helix	0.0041	Preferable	Preferable	-0.8	-1.11	7JJK
LEU	355	A	-64.9	-43.54	A_Helix	0.0044	Preferable	Preferable	5.2	-3.3	7JJK
ALA	356	A	-59.29	-42.33	A_Helix	0.0033	Preferable	Preferable	5.8	-2.37	7JJK
LYS	357	A	-71.36	-32.22	A_Helix	0.0040	Preferable	Preferable	4.7	-2.22	7JJK
ALA	358	A	-77.45	-22.02	A_Helix	0.0037	Preferable	Preferable	5.9	-2.12	7JJK
ASN	359	A	-126.79	63.41	Bend	0.0034	Questionable	Acceptable	5.6	-2.91	7JJK
ALA	361	A	-77.91	-13.74	Turn	0.0041	Preferable	Questionable	-0.9	-1.51	7JJK
ALA	362	A	-88.74	140.13	Bend	0.0037	Preferable	Questionable	2	-1.46	7JJK
ASN	363	A	-83.23	175.33	No_Sec	0.0123	Preferable	Preferable	0.6	-0.62	7JJK
ASN	364	A	-59.2	-39.95	A_Helix	0.0336	Preferable	Preferable	0.4	0.22	7JJK
ALA	365	A	-63.05	-40.44	A_Helix	0.0290	Preferable	Preferable	-0.2	-1.3	7JJK
GLU	366	A	-69.51	-40.6	A_Helix	0.0039	Preferable	Preferable	6.6	-2.03	7JJK
SER	368	A	-60.98	-40.11	A_Helix	0.0042	Preferable	Preferable	8.2	-2.7	7JJK
VAL	369	A	-63.51	-42.69	A_Helix	0.0039	Preferable	Preferable	3.7	-3.5	7JJK
GLY	372	A	-63.46	-40.39	A_Helix	0.0040	Preferable	Preferable	3.9	-1.94	7JJK
GLU	374	A	-65.14	-42.86	A_Helix	0.0045	Preferable	Preferable	6.7	-3.53	7JJK
ASN	376	A	-72.6	-15.52	A_Helix	0.0046	Preferable	Preferable	6	-2.18	7JJK
LYS	377	A	-80.91	-12.78	A_Helix	0.0036	Preferable	Acceptable	3.9	-3.36	7JJK
LEU	378	A	-69.87	144.29	Bend	0.0044	Preferable	Questionable	5.5	-3.04	7JJK
SER	379	A	-72.64	165.6	No_Sec	0.0052	Preferable	Preferable	5	-0.63	7JJK
GLU	380	A	-55.74	-41.95	A_Helix	0.1575	Preferable	Preferable	0.7	0.21	7JJK
GLU	381	A	-63.36	-39.14	A_Helix	0.0076	Preferable	Preferable	0.7	-0.54	7JJK
LYS	384	A	-51.75	-49.93	A_Helix	0.0027	Preferable	Acceptable	5	-3	7JJK
TYR	387	A	-62.76	-42.77	A_Helix	0.0037	Preferable	Preferable	4.5	-3.81	7JJK
ASP	388	A	-59.48	-44.02	A_Helix	0.0038	Preferable	Preferable	7.3	-1.57	7JJK
GLU	389	A	-67.89	-38.85	A_Helix	0.0031	Preferable	Preferable	7.5	-1.85	7JJK
ALA	390	A	-60.26	-36.02	A_Helix	0.0030	Preferable	Preferable	6	-1.67	7JJK
GLN	391	A	-68.14	-39.86	A_Helix	0.0039	Preferable	Preferable	6.8	-1.51	7JJK
LYS	392	A	-62.82	-42.06	A_Helix	0.0036	Preferable	Preferable	4.8	-1	7JJK
ILE	393	A	-60.29	-44.5	A_Helix	0.0045	Preferable	Preferable	6.3	-0.93	7JJK
LYS	394	A	-59.04	-46.83	A_Helix	0.0041	Preferable	Preferable	6.7	-0.44	7JJK
GLU	395	A	-60.63	-40.78	A_Helix	0.0039	Preferable	Preferable	6.7	-0.35	7JJK

Supplementary Table 2. Proline stability classification metrics for tested proline angle and score cutoff criteria.

Score Cutoff	Backbone Angle Proline Classification	Precision	Recall	True Positives	False Positives	True Negatives	False Negatives
-	Preferable	0.20	0.83	104	418	108	22
-	Preferable/Acceptable/ No angle	0.21	0.94	118	434	92	8
ProteinMPNN >= 0.01	-	0.64	0.56	70	40	486	56
ProteinMPNN >= 0.2	-	1.00	0.14	18	0	526	108
Rosetta ΔΔG <= 0.1	-	0.59	0.35	44	31	495	82
ProteinMPNN >= 0.01	Preferable/Acceptable/ No angle	0.64	0.54	68	39	487	58
Rosetta ΔΔG <= 0.1	Preferable/Acceptable/ No angle	0.60	0.34	43	29	497	83

Supplementary Table 3. ProthermDB (2) proline substitutions with Rosetta and ProteinMPNN values from Proscan.

PDB	Amino acid	Residue number	Chain	Experimental ΔΔG (kcal/mol)	Rosetta ΔΔG	ProteinMPNN	-ln(ProteinMPNN)
1c5g	ILE	349	A	0.3	4	0.000	5.600
1g6n	SER	128	A	0.14	7.6	0.004	5.449
1kfw	GLY	253	A	2.6	12.1	0.004	5.573
1kfw	GLY	92	A	-2.2	-1.2	0.061	2.805
1lz1	ALA	47	A	0.1	-0.9	0.052	2.962
1lz1	ASP	91	A	-0.4	0.5	0.018	4.034
1lz1	VAL	110	A	0.5	-0.6	0.434	0.835
1pga	ALA	23	A	0.3	-0.4	0.127	2.067
1pga	ALA	24	A	0.5	-0.4	0.007	4.976
1pga	ALA	48	A	0.7	1.8	0.005	5.339
1pga	ASP	36	A	3.1	6.1	0.003	5.809
1pga	GLY	9	A	2.4	0	0.004	5.655
1pga	LYS	10	A	0.2	8.9	0.011	4.501
1pga	THR	25	A	2.8	8.6	0.003	5.878
1pga	THR	2	A	2.7	1	0.003	5.952
1pga	VAL	21	A	-0.5	-0.6	0.099	2.311
1pga	VAL	29	A	3.5	3.9	0.003	5.714
1rop	ASP	30	A	1.6	2.4	0.004	5.547
1rtp	ALA	21	1	-1.92	-1	0.588	0.531
1rtp	HIS	26	1	1.25	0.3	0.336	1.090
2lzm	ALA	74	A	4.5	7.8	0.005	5.360
2lzm	ASP	72	A	2.7	6.2	0.004	5.521
2lzm	GLN	69	A	2.9	5	0.003	5.776
2lzm	ALA	82	A	-0.8	-0.8	0.163	1.816
2lzm	ARG	96	A	5.5	11.7	0.003	5.745
2rn2	HIS	62	A	-1.1	-0.5	0.290	1.239
2rn2	ALA	52	A	1.6	-0.5	0.004	5.627

References

1. Tsuboyama, K., Dauparas, J., Chen, J., Laine, E., Mohseni Behbahani, Y., Weinstein, J.J., Mangan, N.M., Ovchinnikov, S. and Rocklin, G.J. (2023) Mega-scale experimental analysis of protein folding stability in biology and design. *Nature*, **620**, 434-444.
2. Nikam, R., Kulandaisamy, A., Harini, K., Sharma, D. and Gromiha, M.M. (2021) ProThermDB: thermodynamic database for proteins and mutants revisited after 15 years. *Nucleic Acids Res*, **49**, D420-D424.
